# Milk Microbiota: What Are We Exactly Talking About?

**DOI:** 10.3389/fmicb.2020.00060

**Published:** 2020-02-14

**Authors:** Georgios Oikonomou, Maria Filippa Addis, Christophe Chassard, Maria Elena Fatima Nader-Macias, I. Grant, Celine Delbès, Cristina Inés Bogni, Yves Le Loir, Sergine Even

**Affiliations:** ^1^Institute of Veterinary Science, University of Liverpool, Neston, United Kingdom; ^2^Dipartimento di Medicina Veterinaria, Università degli Studi di Milano, Milan, Italy; ^3^Université Clermont Auvergne, INRAE, UMRF, Aurillac, France; ^4^CERELA-CONICET, San Miguel de Tucumán, Argentina; ^5^Departamento de Microbiología e Inmunología, Facultad de Ciencias Exactas, Físico-Químicas y Naturales, Universidad Nacional de Río Cuarto, Río Cuarto, Argentina; ^6^STLO, UMR 1253, INRAE, Agrocampus Ouest, Rennes, France

**Keywords:** milk microbiota, mammary gland, metagenomics, microbial community, enteromammary pathway, offspring gastrointestinal microbiota

## Abstract

The development of powerful sequencing techniques has allowed, albeit with some biases, the identification and inventory of complex microbial communities that inhabit different body sites or body fluids, some of which were previously considered sterile. Notably, milk is now considered to host a complex microbial community with great diversity. Milk microbiota is now well documented in various hosts. Based on the growing literature on this microbial community, we address here the question of what milk microbiota is. We summarize and compare the microbial composition of milk in humans and in ruminants and address the existence of a putative core milk microbiota. We discuss the factors that contribute to shape the milk microbiota or affect its composition, including host and environmental factors as well as methodological factors, such as the sampling and sequencing techniques, which likely introduce distortion in milk microbiota analysis. The roles that milk microbiota are likely to play in the mother and offspring physiology and health are presented together with recent data on the hypothesis of an enteromammary pathway. At last, this fascinating field raises a series of questions, which are listed and commented here and which open new research avenues.

## Introduction

The development of high-throughput sequencing techniques (including second- and third-generation sequencing and combinations thereof) has dramatically reduced the cost and enhanced the efficiency and the accuracy of DNA sequencing, enabling the rise of metagenomic or metataxonomic investigations in numerous ecosystems. These techniques suggest the existence of microbial communities in unexpected niches so far, including body sites and fluids that had long been considered sterile when healthy (i.e. not infected), such as chick cecum *in ovo* (Kizerwetter-Świda and Binek, 2016), meconium (Borghi et al., 2018), respiratory tract (Zeineldin M. et al., 2017; Zeineldin M. M. et al., 2017), and milk.

Recent work indeed suggested that human milk, beyond providing neonates with adequate nutrients, supplies microbes to the newborn infants’ gastrointestinal tract (GIT) during their early and critical period of development (Jeurink et al., 2013; Jost et al., 2015; Rautava, 2016; Murphy et al., 2017; Pärnänen et al., 2018). Over the last decade, reports of viable bacteria in milk produced by healthy women introduced a debate on the (lack of) sterility of human milk, apart from infection, and suggested that bacteria present in human milk not only originate from the skin or other environmental sources but are instead ubiquitously present in milk produced by healthy women (Fernández et al., 2013; Jeurink et al., 2013; Jost et al., 2014; Murphy et al., 2017).

This is also the case for bovine milk. At the time this community is being explored, several questions and sometimes controversies are raised concerning its origin, its role and even its existence (Jost et al., 2015; Addis et al., 2016; Rainard, 2017; Derakhshani et al., 2018a; Metzger et al., 2018b). It thus appeared relevant to us to attempt to describe the research that has already been done on the milk microbiota and what is exactly known. What are the lessons from this first set of studies, and what was the purpose of these studies? Which conclusions can be drawn? What are the limitations we need to consider? In this review, we will include research on the milk and the mammary gland microbiota. Studies on both humans and ruminants are included in a cross-species approach. We will end with future prospects: which questions need further investigations? Which opportunities does the existence of this milk microbiota offer to address human and animal health issues?

## Milk Microbiota: Current Studies and Limits

Prior to any discussion on milk microbiota, an overview on the ways milk microbial community has been explored is necessary to clarify what is exactly known so far and what the limits of these studies are. In agreement with the definition proposed by Marchesi and Ravel (2015), milk microbiota refers to the assemblage of microorganisms present in milk. By extension, microorganisms associated with the mammary gland or teat will be included in this review.

**FIGURE 1:**
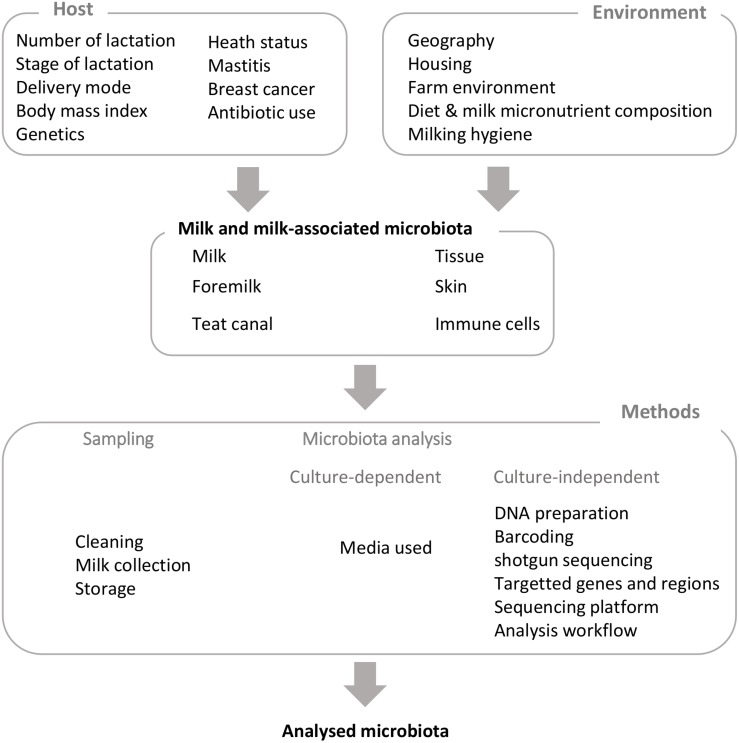
Factors influencing milk and milk-associated microbiota and technical biases.

The milk microbiota has been mostly investigated in women (Hunt et al., 2011; Jost et al., 2013; Fitzstevens et al., 2016) and in cows (Oikonomou et al., 2014; Addis et al., 2016; Falentin et al., 2016); some studies were also conducted in other mammals such as goats, sheep, donkeys, buffalo, water deer, reindeer, or mice (Quigley et al., 2013; McInnis et al., 2015; Treven et al., 2015; Catozzi et al., 2017; Li et al., 2017; Soto Del Rio et al., 2017; Supplementary Table S1). Most studies on milk microbiota have investigated milk collected by manual expression, generally following thorough cleaning of nipples or teats (Oikonomou et al., 2012; Jost et al., 2013; Boix-Amoròs et al., 2016) (Figure 1). However, a recent study investigated the microbiota composition of human milk collected in a non-aseptic environment, corresponding to “Breastfeeding-associated microbiota of human milk” rather than human milk microbiota (Simpson et al., 2018). Most studies on milk microbiota have used mature milk, but few have investigated colostrum microbiota in human and bovines (Aakko et al., 2017; Lima et al., 2017; Toscano et al., 2017; Derakhshani et al., 2018c). Some studies considered milk collected from only one nipple or teat, whereas others considered pooled milk (Dolci et al., 2014; Chaves Lopez et al., 2016). Bacteria have been shown to be present in milk in a free-living, “planktonic” state, but they can also be associated with immune cells. In most of the above-mentioned studies, milk microbiota has been explored once milk has been expressed, or in other words, outside of the mammary gland. Whether microbiota can be associated with milk inside the mammary gland remains to be determined. A recent study based on metataxonomics reported the existence of microbiota associated with bovine milk collected by direct sampling into the cistern using a needle and vacuum tube, and this was observed for healthy quarters of the mammary gland with low somatic cell counts (<100,000 cells/ml) (Metzger et al., 2018a). Its composition differed from the microbiota associated with milk collected by conventional septic techniques. However, all these cistern milk samples were culture negative, suggesting that bacteria were either dead or viable but non-cultivable in the growth conditions used. This study was done on a limited number of samples, and further research is required to clarify whether live microorganisms are present in milk inside a healthy mammary gland. Apart from milk, a limited number of studies have explored the microbiota associated to the bovine teat skin or teat apex (Gill et al., 2006; Braem et al., 2012; Verdier-Metz et al., 2012; Frétin et al., 2018), or the internal teat by sampling foremilk and swabbing the teat canal (Falentin et al., 2016; Derakhshani et al., 2018c). Similarly, only few reports are available about the microbiota associated with the mammary gland tissues in human and mouse (Urbaniak et al., 2014; Treven et al., 2015).

Milk microbiota exploration relies on both culture-dependent and culture-independent approaches, including sequencing of 16S rRNA clone libraries (Gill et al., 2006; Verdier-Metz et al., 2012) and, in the last 10 years, metataxonomics, based on 16S rRNA gene amplicon sequencing (Supplementary Tables S1–S4). Methods used to explore milk microbiota are likely to introduce major bias in the taxonomic profile of the milk microbial community. For example, culture-dependent approaches only target cultivable bacteria. Isolates will strongly depend on the media used, sample storage, and growth conditions. On the contrary, molecular approaches such as metataxonomics detect DNA from all the bacteria present in a sample, alive or not. Considering this major bias of molecular approaches, methods have been developed to selectively amplify DNA of the intact fraction of the population, based on the use of propidium monoazide (Erkus et al., 2016). Moreover, even within these metataxonomics-based studies, several factors will introduce some variability: the milk fraction used, the lysis method (enzymatic, mechanical, or both), the purification kit, the target gene (or region) used to generate amplicons, the number of PCR cycles, PCR primers, and also the sequencing platform (Tremblay et al., 2015; Fouhy et al., 2016; Knudsen et al., 2016; Castelino et al., 2017; Rintala et al., 2017; Lima et al., 2018; Metzger et al., 2018a; Supplementary Tables S1–S4). This results in variation in overall coverage and in differential amplification efficiencies between taxa as illustrated by *in silico* PCR on a 16S rRNA gene database (Klindworth et al., 2013) and experimental validation on mock communities (Tremblay et al., 2015; Fouhy et al., 2016). It is also important to mention here that these PCR-based approaches can amplify very low levels of DNA from contaminants, and therefore, the inclusion of negative control samples at each step is crucial (e.g. DNA extraction-negative and PCR-negative control samples) (Avershina et al., 2018; Pollock et al., 2018); this unfortunately was not the case for a proportion of the published human or bovine milk microbiota studies. Workflows used for data analysis are also likely to introduce some variability in bacterial community (Escudié et al., 2018). A major limitation of metataxonomic studies is also that these approaches allow description of microbial communities mainly at the genus level or higher taxonomic levels, thus precluding diversity exploration at the species or even strain levels. A higher taxonomic resolution of these communities can be achieved by using shotgun metagenomic approaches. Few studies on milk microbiota using shotgun metagenomic approaches are now available (Bhatt et al., 2012; Jiménez et al., 2015; Pärnänen et al., 2018; Hoque et al., 2019). These studies also allowed exploration of archaeal, fungal, and viral communities in addition to bacterial communities. Besides, they gave access to a functional profiling of these microbial communities, including data on microbial metabolism, virulence, or antibiotic resistance (Hoque et al., 2019). Intermediate approaches between metataxonomic and shotgun metagenomic approaches have also been undertaken, based on reduced metagenomic sequencing, allowing a deeper taxonomic characterization at the species and even strain levels (Avershina et al., 2018).

Hence, although the presence of microorganisms in milk is supported by a large set of investigations all over the world in different hosts, one should keep in mind samplings and methods that have been used and thus limits to conclusions that can be drawn.

## A Core Milk Microbiota Universally Shared Between Hosts?

A number of studies have characterized the milk microbiota mainly in human and cows and mainly using culture-independent approaches; all of them describe a complex and diverse community (Cabrera-Rubio et al., 2012; Oikonomou et al., 2014; Addis et al., 2016; Murphy et al., 2017; Derakhshani et al., 2018c). As an illustration, Murphy et al. studied 10 mother–infant pairs and identified over 207 genera in human milk microbiota. In this study, milk microbiota exhibited a higher diversity compared to infant feces (Murphy et al., 2017). On the contrary, Pärnänen et al. reported a lower diversity in milk compared to infant feces as determined by shotgun metagenomics (Pärnänen et al., 2018). The low microbial DNA abundance and the lack of amplification step may account for such discrepancy. As suggested by these authors, limitations in the direct sequencing of milk microbial DNA may account for a lower sequencing depth, making it more difficult to observe low-abundance taxa (Pärnänen et al., 2018). Similarly, a complex microbial community was reported in cow milk by Hoque et al. (2019), with 146 bacterial strains identified in healthy milk by shotgun metagenomic sequencing.

**FIGURE 2:**
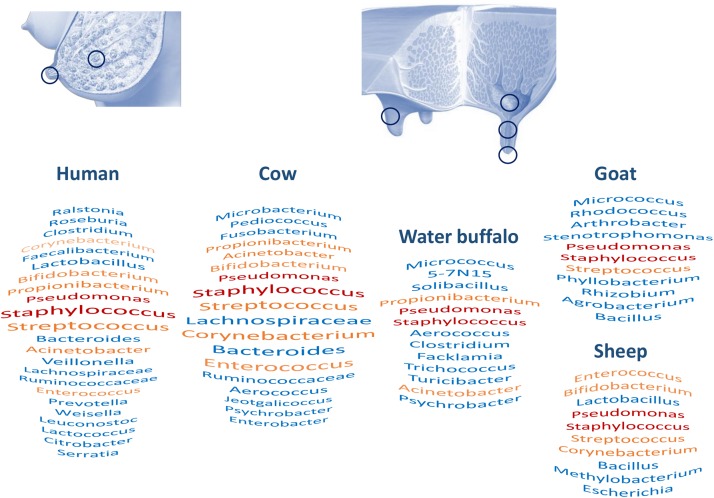
Milk and milk-associated microbiota in humans and animals: sampling sites and major taxa. Figure based on Supplementary Tables S1–S4. Red and orange taxa are shared between all human and animal species or present in three species out of five, respectively. For humans and bovines, taxa size reveals citation frequency.

An overview of studies on milk microbiota clearly points out common taxa between human and cow milk (Supplementary Tables S1–S4 and Figure 2): *Staphylococcus*, *Streptococcus*, *Pseudomonas*, *Bifidobacterium*, *Propionibacterium*, *Bacteroides*, *Corynebacterium*, and *Enterococcus* are among the most cited dominant taxa in studies on both human and bovine milk microbiota (Oikonomou et al., 2014; Jiménez et al., 2015; Addis et al., 2016; Boix-Amoròs et al., 2016; Urbaniak et al., 2016; Murphy et al., 2017; Derakhshani et al., 2018a). On the other hand, Metzger et al. (2018b) did suggest that the detection of *Pseudomonas* could be attributed to contamination issues as it was one of the genera they found to be abundant in their negative control samples. Several metataxonomic studies have proposed the existence of a core human milk microbiota (Figure 2; Hunt et al., 2011; Murphy et al., 2017). Similar milk bacterial profiles were obtained using a shotgun metagenomic approach (Jiménez et al., 2015; Pärnänen et al., 2018). Such approaches also allowed them to describe the presence of fungal, protozoal, and viral DNA in the same milk samples. Human milk microbiota diversity has also been supported by culture-dependent approaches, which beyond the dominant genera *Staphylococcus*, *Streptococcus*, and *Propionibacterium* allowed the isolation of members of *Bifidobacterium*, *Rothia*, *Enterococcus*, *Lactobacillus*, or even obligate anaerobes such as *Veillonella* (Perez et al., 2007; Jost et al., 2013). Milk microbiota has also been investigated in other animals, including donkey, goat, sheep, water deer, reindeer, and water buffalo, although the number of studies is limited compared to that of studies of human and cow milk (Supplementary Table S1), showing some overlap with human and cow milk microbiota (Figure 2; Castro et al., 2011; McInnis et al., 2015; Catozzi et al., 2017; Li et al., 2017; Soto Del Rio et al., 2017; Esteban-Blanco et al., 2019). Nevertheless, significant differences have been reported in the milk bacterial communities of different ruminants, such as water deer, reindeer, and goat, suggesting host microbial adaptation, although influence of environment and herd management should not be excluded (Li et al., 2017).

In conclusion, this comparative analysis of milk microbiota associated with different hosts points out common taxa, including the *Staphylococcus* and *Streptococcus* genera (Supplementary Tables S1–S4 and Figure 2). A systematic review of the human milk microbiota had already identified *Staphylococcus* and *Streptococcus* as universally predominant in human milk (Fitzstevens et al., 2016). A comparison between hosts invites us to consider the existence of a core milk microbiota universally shared between hosts and opens questions about its role. Nevertheless, additional investigations with better harmonization of methods will be necessary to clearly address this question; that may include a deeper characterization of taxa, at the species level, and exploration of the functional profiles of these microbial communities.

## Origins of Milk Microbial Community

The question regarding the genesis of the milk microbiota remains largely unanswered. Bacteria present in expressed milk are likely to come from contamination through bacterial exposure of the breast (or udder) during and in between nursing as well as from endogenous sources via a yet hypothetical enteromammary pathway, as reviewed for human (Jost et al., 2015) and bovine milk (Addis et al., 2016; Supplementary Table S2). It has long been considered that the milk microbiota resulted from external contamination by the mother’s skin or the oral cavity of the baby. Similar observations were made regarding bovine milk and teat skin (Doyle et al., 2017). The presence of members of the oral cavity in the milk microbiota has been widely reported (Cabrera-Rubio et al., 2012; Murphy et al., 2017). Some retrograde flow back into the mammary ducts can occur during suckling or mechanical milking (Ramsay et al., 2004). Williams et al. (2019) estimated the infant oral microbiota contributed to milk microbiota to be ∼21% at day 2 and 66% at month 5. Of course, milk can also provide bacteria to the oral cavity of the baby or the offspring. As mentioned above, human milk contains species of genera such as *Propionibacterium* sp., *Staphylococcus* sp., or *Corynebacterium* sp., which are usual inhabitants of the adult skin ecosystem. Beyond these common phylotypes, extensive differences are reported between the compositions of milk and skin bacterial communities (Hunt et al., 2011; Cabrera-Rubio et al., 2012). For instance, human milk contains bifidobacteria and lactobacilli that are not encountered in the breast skin microbiota (Martín et al., 2009). For a given *Enterococcus* or *Lactobacillus* species in the same host, the milk isolates are genotypically different from those isolated from the host skin (Martín et al., 2003). In a recent study, the milk, vaginal, and fecal microbiota were compared in women just before and after giving birth, showing low redundancy in terms of bacterial species (Avershina et al., 2018). Moreover, milk samples were significantly more similar to either vaginal or fecal samples from the same mother than among different mothers. Such observation is in line with the translocation of maternal microbiota to the milk (Avershina et al., 2018).

With milk now being regarded as an inoculum for the infant GIT, the existence of an enteromammary route has been explored. Although milk and maternal feces microbiota are distinct, a strong correlation has been reported between them (Williams et al., 2019). The isolation of a common strain of the obligate anaerobe *Bifidobacterium longum* from maternal and neonate feces as well as from milk supports the hypothesis of an enteromammary route (Jost et al., 2014). Viable bacteria are found in the mammary tissue of women who have never breastfed, suggesting that the mammary gland itself may be a source of bacteria for milk (Urbaniak et al., 2014). The possibility of an active “sampling” and translocation of bacteria from the lumen of the mother’s GIT to the mammary glands has also been suggested (Perez et al., 2007; Donnet-Hughes et al., 2010). This has been supported by a study carried out in a lactating mouse model where, during pregnancy and lactation, bacterial translocation from the GIT increases and dendritic cells loaded with gastrointestinal bacteria are found in the mammary tissue (Perez et al., 2007). In women, the analysis of milk and peripheral blood samples aseptically collected from healthy breastfeeding women and feces from mothers and infants revealed common bacterial signatures as determined by TTGE, suggesting that immune cells might be able to transport intestinal bacteria or, at least, bacterial components from the GIT to the mammary gland and infant GIT (Perez et al., 2007). In lactating cows, indication of bacterial transfer from the intestinal lumen to the mammary gland has also recently been suggested (Young et al., 2015). Samples of blood and milk leukocytes and feces were collected from healthy lactating cows, and their bacterial composition was investigated using a metataxonomic approach. Of note, in this study, a catheter was used to collect milk and avoid any contamination by bacteria of external source. A few bacterial operational taxonomic units (OTUs) corresponding to *Bifidobacterium* and *Ruminococcus* genera and the *Peptostreptococcaceae* family were found common to the three sample types. However, whether these common OTUs corresponded to the same strains and whether these strains were viable were not established. Additional investigations are required to address the possibility of transfer of viable bacteria from the intestinal tract to milk in both women and cows via an enteromammary route, all the more since these intestinal bacteria would have to overcome several immunological firewalls and unexpectedly deceive the immune system after translocation in order to reach the mammary gland (Rainard, 2017). Besides, while the disruption of the mammary gland epithelium integrity, as observed during mastitis, may facilitate translocation of bacteria through the epithelium (Schwarz et al., 2018), translocation of intestinal bacteria through an intact mammary gland epithelium remains to be established. Nevertheless, these studies at least suggest the transfer to milk and infants, through this enteromammary route, of bacterial components that may contribute to programming the neonatal immune system, allowing discrimination between pathogens and commensal organisms (Perez et al., 2007).

## Factors Influencing Milk Microbiota Composition

Besides the biases introduced by sampling and methods, several host and environmental factors have been reported to influence milk microbiota composition in human and cows (Figure 1 and Supplementary Table S3). Of note, most factors investigated for human and bovines are not exactly the same and, as expected, are in relation with management practices for cows (see Derakhshani et al., 2018a, for a recent review of factors that potentially influence bovine mammary microbiota). Among host factors affecting milk microbiota, several studies have reported a relation between milk microbiota and mother’s health, with special attention to mammary gland infections. While mother’s health could be seen, at first glance, as a factor influencing milk microbiota composition, studies investigating links between mammary gland health and milk microbiota suggest a more complex relation far from a one-way one. This point will be addressed in a specific part of this review dedicated to both mother and infant health.

### Host Factors

It has been shown that the composition of human milk changes with time, between colostrum, transition, and mature milk with, notably, an increase of genera commonly found in the oral cavity (Cabrera-Rubio et al., 2012; Boix-Amoròs et al., 2016; Murphy et al., 2017). The authors suggest that this evolution is notably due to the individual crosstalk between mother and infant through retrograde flow (Murphy et al., 2017; Moossavi et al., 2019). Nevertheless, significant changes with time are somehow difficult to detect, in relation to strong interindividual variations. Hence, Williams et al. (2017) suggested that the human milk microbiota was relatively constant over time. Crosstalk between cow and calf is very limited in dairy cows due to early separation between both. Nevertheless, bovine milk microbiota was also found to vary with time, with both the stage and the number of lactations. Colostrum was recently shown to harbor a complex and diverse microbiota, whose richness was significantly higher in primiparous cows compared to multiparous cows (Lima et al., 2017). Taxonomic profiles and alpha diversity were also related to the stage of lactation and varied during the first week of lactation (Derakhshani et al., 2018b). Differences were also observed in clinically healthy Holstein dairy cows between microbiota of the teat canal and mammary secretions (milk or colostrum) at the time of drying off and immediately after calving (Derakhshani et al., 2018c).

In addition, differences were found in human milk microbiota in relation with the delivery mode (Cabrera-Rubio et al., 2012; Khodayar-Pardo et al., 2014; Hermansson et al., 2019). A greater bacterial diversity was found in milk from women who delivered vaginally, compared to women who delivered through C-section. Interestingly, this was not the case for emergency C-section, which was similar to that of vaginal delivery (Cabrera-Rubio et al., 2012). C-section could indeed affect the infant microbiota and through this affect the milk one. On the other hand, Urbaniak et al. (2016) found no evidence of statistically significant differences between the microbiota profiles of milk in preterm and term, C-section (elective and emergency), and vaginal delivery, and even those in male and female infants. Such studies are scarce in bovine herds although implications for this in bovine medicine would be fascinating, namely, for cattle that have a genetic predisposition to C-sections such as certain beef breeds.

Human milk microbiota composition was also found to be related to additional host factors such as the body mass (Cabrera-Rubio et al., 2012; Williams et al., 2017). However, contradictory results have been obtained on the relation between the mother’s body mass index and milk microbiota diversity (Davé et al., 2016). The authors suggested that these discrepancies could be related to the pronounced differences in terms of geographical location, socioeconomic status, diet, and ethnicity between women enrolled in the different study groups. Results obtained on 393 mother–infants dyads of the CHILD cohort support the influence of the body mass index, parity, mode of delivery, and breastfeeding practices (at breast versus pumped) (Moossavi et al., 2019). In bovines, several host-associated factors are likely to influence milk and mammary gland microbiota such as physiological parameters and anatomical characteristics of the teat and genetic traits (Derakhshani et al., 2018a).

### Environmental Factors

Geography has been shown to play a role in microbiota patterns. An important study on geographical influence on the microbiota showed significant intercountry differences between milk microbiota of Spanish, Finnish, South African, and Chinese women (Kumar et al., 2016). However, these geographical differences could be due to several factors, such as environment, diet, or even genetics. In bovines, while the influence of “geography” *per se* has not been studied, milk microbiota was found to be related to the farm environment and management practices (Doyle et al., 2017; Derakhshani et al., 2018a; Frétin et al., 2018; Metzger et al., 2018a). Metzger et al. (2018a) reported a relation between the bedding material and the bovine milk microbiota profiles. Likewise, milking practices including premilking teat preparation treatment were reported to affect milk microbiota (Doyle et al., 2017).

Diet has also been reported to affect milk microbiota in both humans and cows. The human milk bacterial community was shown to correlate with specific fatty acid profiles, suggesting a relationship between diet and milk composition (Kumar et al., 2016). Such relation with maternal nutrient intake was also reported by Williams et al. (2017), who observed a relation between the intake of saturated and monounsaturated fatty acids, carbohydrates, and proteins and the relative abundance of several taxa in milk bacterial community. Likewise, in cows, Zhang et al. (2015) also suggested a possible effect of diet on the bovine milk microbiota. A high-concentrate diet in this study was associated with higher abundance of some mastitis-causing pathogens in milk.

Among factors that have been shown to influence microbiota in general and be deleterious to microbiota diversity, antibiotics play a particular role due to a direct impact on the microbial community. The influence of antibiotics has been especially documented for cow milk. Among common herd management practices, antibiotics and teat sealant are currently administered at the dry period (i.e. period between two lactations) in relation to an increased risk of infections during this period, in order to decrease or eliminate subclinical infections and to prevent new infections at the next lactation (Bradley and Green, 2004). Such DCT was suspected to affect and disturb the microbiota of healthy mammary gland. Bonsaglia et al. (2017) evaluated this hypothesis by determining the effect of DCT with teat sealant alone or with antibiotics (ceftiofur hydrochloride) on non-mastitic cows. They found that omitting antibiotics from DCT has no effect on the milk microbiota at dry off and 7 days postpartum in the subsequent lactation. The authors suggest that bacterial communities are dynamic and that the antimicrobially induced disturbance of milk microbiota is reversed by the time that cows start a new lactation. Likewise, antimicrobial DCT combining penicillin G and novobiocin in internal teat sealant showed that a considerable number of bacterial genera, including those commonly regarded as mastitis pathogens, were common between the pre-DCT and postpartum microbiota, suggesting a high resilience of the mammary microbiota after exposure to antimicrobials during the dry period (Derakhshani et al., 2018c). Another possible explanation of these findings could be that a large proportion of what is described as the milk microbiota is DNA from bacteria that are already dead and will therefore not be affected by antibiotic therapy.

This overview of factors influencing the milk microbiota in both humans and cows invites us to consider it as a dynamic community which can be shaped by several factors, including host factors such as mode of delivery, mother physiological parameters, lactation stage and parity, genetic traits, and infant mouth microbiota but also environmental factors such as diet, geography, management practices, milking hygiene, and health management (antibiotic use). While some of the above-mentioned factors may be used to drive microbiota toward a targeted composition, others are inherent to the host and, as such, have to be considered. A better understanding of factors influencing milk microbiota composition, their relative importance, their short- and long-term effects, and the resilience of this microbiota to perturbations is undoubtedly a challenge but also a prerequisite to further shape the microbiota toward compositions beneficial to mother and infant health.

## Milk Microbiota: Role for Mother and Infant Health

### Milk Microbiota and Mother’s Health

Milk microbiota composition has been associated with mother’s health, especially mammary gland health. For instance, bacterial dysbiosis has been described in lactating mothers suffering from breast pain and/or mastitis (Jimenez et al., 2008; Jiménez et al., 2015; Maldonado-Lobón et al., 2015; Mediano et al., 2017). In mastitis cases, the milk microbiota reflects a loss of bacterial diversity and a high increase in the abundance of the sequences related to the presumptive etiological agents such as *Staphylococcus aureus* and *Staphylococcus epidermidis* in acute and subacute mastitis, respectively (Jiménez et al., 2015; Patel et al., 2017). Likewise, in bovine, both clinical and subclinical intramammary infections are associated with changes in milk microbiota (Oikonomou et al., 2012, 2014; Kuehn et al., 2013; Falentin et al., 2016; Lima et al., 2017). Some studies reported a loss of diversity in unhealthy milk or teat (Braem et al., 2012; Oikonomou et al., 2012; Falentin et al., 2016; Ganda et al., 2017), whereas another observed a higher number of bacterial strains in mastitic milk (Hoque et al., 2019). Most studies have been carried out to better understand bacterial ecology at the time of infection (Oikonomou et al., 2012; Kuehn et al., 2013). This is of particular interest as pathogen(s) responsible for mastitis is not identified by culture-dependent methods in 10–40% of clinical mastitis cases (Makovec and Ruegg, 2003). In a longitudinal study, Ganda et al. (2017) described the bovine milk microbiota before, during, and after experimentally induced mastitis with *Escherichia coli*. Interestingly, milk microbiota was transiently affected by the *E. coli* infection but returned to the initial composition prior to *E. coli* infection, in both quarters treated with ceftiofur, a third-generation cephalosporin, and untreated quarters, suggesting resilience of milk microbiota to infection. While the above-mentioned studies explore changes in bacterial communities at the time of infection, a few additional studies have investigated the relationship between the milk or the mammary gland microbiota and health prior to or following an infectious episode (Falentin et al., 2016; Lima et al., 2017). The teat microbiota was investigated in quarters with different history of mastitis (Falentin et al., 2016). Healthy quarters showed a higher diversity compared to those with a history of mastitis in previous lactations and exhibited a different bacterial profile, notably within Firmicutes. These microbiota alterations may have resulted from the infection and or antibiotic use, or alteration of microbiota may have been present prior to infection. In agreement with this latest hypothesis, Lima et al. (2017) were able to separate bacterial profiles of colostrum between quarters that developed or did not develop mastitis in the first 30 days postpartum. Interestingly, a lower diversity was observed in the microbiota of primiparous quarters that developed clinical mastitis in the first month of lactation, compared to quarters with no clinical mastitis. Taxonomic markers of health status could be defined in primiparous cows, but such discrimination was not conserved in multiparous cows. Whether changes in milk microbiota in relation to intramammary infections are just a consequence of the infection or contributed to the onset of infection is still a matter of debate. This is also the case for several health disorders in both humans and animals as relation of causality would require a kinetic exploration of microbiota including time points prior to the onset of disorder.

Relations between milk microbiota and mother’s health have also been reported in human for diseases targeting distant anatomic sites. Microbiota was impacted in women suffering from celiac disease (Olivares et al., 2014) and contained less bifidobacteria, although this may be related to the disease itself or the modified diet. Changes in human milk microbiota composition associated with infections by viruses or with medical treatments were also documented. Milk microbiota diversity was impacted in HIV-infected women compared to healthy women from the same area (González et al., 2013). Women following chemotherapy also exhibited substantial modifications of their milk microbiota.

Finally, milk microbiota can be influenced by mother’s health in terms of transmission of pathogens. Vertical transfers of viruses (dengue) or bacterial pathogens (*Salmonella* and *Streptococci* B) through milk are reported in medical case studies (Qutaishat et al., 2003; Wang et al., 2007; Cooke et al., 2009; Barthel et al., 2013; Vilca et al., 2015) showing that the mother’s health status can directly affect infant safety through breastfeeding. Most of these studies did not investigate milk microbiota, to confirm or refute a bacterial dysbiosis, as seen in HIV-infected women. If direct transfers from mother to infant independently on the milk microbiota are possible, further research is warranted to evaluate the role of milk microbiota dysbiosis for pathogen transfer according to the mother’s health status.

### The Milk Microbiota and Infant Health

Milk is more than a mere source of nutrients. Beyond its role in nutrition, milk has several and complex roles in the development of the offspring. It indeed contains non-nutritive biologically active compounds such as immunoglobulins, oligosaccharides, growth factors, epithelial and immune cells, inhibitory systems, and DNA. It thus likely plays a role in the immunity of the newborn and in the maturation of the digestive tract and of the digestive immune system. It also contains bacteria, which may serve as inoculum and play a role in the colonization and homeostasis of the gastrointestinal microbiota.

The infant gastrointestinal microbiota colonization has long been regarded as the sole result of environmental contributions, resulting from the vaginal passage, the mother–child skin contacts, and newborn’s environment. Human milk microbiota is now also considered as a reservoir of microbes for gastrointestinal colonization in newborns (Jost et al., 2014; Williams et al., 2019). This is supported by the significant overlap between infant feces and mother milk bacterial compositions. Shared genera between mother’s milk and infant feces accounted for 70–88% of the relative abundance of fecal microbiota (Murphy et al., 2017). Contribution of milk microbiota to infant gastrointestinal microbiota was also pointed out by Williams et al. (2019), who estimated a direct contribution of ∼4.9% and suggested additional contribution through a probable effect on the microbiota present in the stomach, small intestine, and upper large intestine. Likewise, in a metagenomic study, Pärnänen et al. (2018) reported that 76% of the species found in milk were present in the infant’s GIT. Their results also pointed out the similarity of antibiotic resistance genes and mobile genetic elements between related mothers’ GIT and milk and infants’ GIT (Pärnänen et al., 2018). Bacteria commonly found in human milk are part of the predominant taxa found in newborns during the first weeks of life (Jost et al., 2012, 2013). Many studies have demonstrated an early vertical transfer of both facultative and strict anaerobes (*Bifidobacterium* species and potentially others) from human milk to the infant GIT (Jost et al., 2014; Duranti et al., 2017). In agreement with this vertical transfer from mother’s milk to infant gut, overlap of bacterial composition was observed between mother’s milk and infant’s mouth microbiota (Biagi et al., 2017). Of note, we cannot exclude that milk microbiota contributes to other infant’s microbiota such as the upper respiratory tract through the oral cavity, but this will need additional investigations.

Human milk microbes are part of the pioneer colonizers initiating the gastrointestinal microbiota establishment and structuring a unique microbial ecosystem responsible for GIT health and key functions (Jost et al., 2014). As facultative anaerobes, *Staphylococcus*, *Streptococcus*, and *Lactobacillus* species are able to early colonize the GIT, reducing the local environment by consuming O_2_ and contributing to the generation of a complete anaerobic system favorable for a subsequent implanting of strict anaerobes (Cerdó et al., 2018; Nogacka et al., 2018). Transfer of anaerobic microbes such as aerotolerant *Bifidobacterium* species was confirmed by multiple studies (Duranti et al., 2017). Transfer of other strict anaerobes including *Bacteroides* species or *Veillonella* species has been suggested by molecular approaches but should be further confirmed with culture methods.

Interestingly, most of human milk bacteria are involved in lactate metabolism, for either lactate production (*Staphylococcus*, *Streptococcus*, and *Lactobacillus*) or lactate utilization (*Propionibacterium* and *Veillonella*). Presence of both functional groups should contribute to the establishment of an effective trophic chain avoiding lactate accumulation and any negative impact such as acidosis and further toxicity for the brain in infants (Kang et al., 2006; Pham et al., 2016, 2017). More recently, the presence of fungi including *Saccharomyces* species has been reported in human milk samples (Boix-Amorós et al., 2017). Yeasts are therefore part of the lactate-utilizing community of human milk and should further colonize the infant intestine, contributing to lactate disappearance. *Lactobacillus* and *Bifidobacterium* species are also known to metabolize human milk oligosaccharides (HMO), thus promoting their growth. Bifidobacteria from milk are genetically adapted to HMO utilization (Duranti et al., 2017). Metabolic cross-feeding interactions between commensal gastrointestinal microbes during HMO metabolism is an important driver of gastrointestinal health and immunity by promoting gastrointestinal colonization by human milk microbiota (Jost et al., 2015; Lewis et al., 2015). Those microbes can directly interact with the immune system and contribute to its maturation during the first month of life.

## Discussion: Open Questions and Future Directions

There are numerous questions that still need to be answered before we can take actions for influencing the milk microbiota.

### Does a Live Microbiota Exist in Milk?

One of the main arguments against the possible presence of a complex healthy milk microbiota is that most studies on milk microbiota rely on metataxonomics whereas only few studies are based on culture-dependent approaches (Jost et al., 2013). DNA amplification techniques may highlight the presence of non-viable microorganisms including those that could be present within phagocytes (Rainard, 2017). A comparison between the total bacterial population as determined by culture-dependent and culture-independent methods points out significant discrepancies. Boix-Amorós et al. used qPCR in order to quantify the bacterial load in milk samples obtained from healthy mothers and reported a median bacterial load of 10^6^ bacterial cells/ml; a large proportion of these bacterial cells were shown to exist in milk in a free-living state and not to be associated with human cells (Boix-Amoròs et al., 2016). Likewise, using qPCR, Falentin et al. (2016) reported a bacterial load of the internal cow teat microbiota of between 10^4^ and 10^5^ bacterial cells/ml. Culture-dependent characterization of human milk rather revealed a bacterial count between 10^2^ and 10^4^ bacterial cells/ml, suggesting that a part of the milk microbiota, as determined by molecular approaches, may correspond to non-viable (or non-cultivable) bacteria or that only a fraction of this bacterial community has been isolated so far (Jost et al., 2013). In agreement with this, structures of milk bacterial communities differ between culture-dependent and culture-independent methods. Jost et al. reported that 90% of isolated strains corresponded to *Staphylococcus*, *Streptococcus*, and *Propionibacterium* whereas their relative abundance using pyrosequencing was 23.7%. Nevertheless, obligate anaerobes, including *Bifidobacterium* and *Veillonella*, have also been isolated, suggesting that the living part of milk microbiota may not have been fully or properly explored so far. The biodiversity of the milk microbiota (Kuehn et al., 2013) as well as its resilience following infection or antibiotic treatment (Ganda et al., 2016; Bonsaglia et al., 2017; Derakhshani et al., 2018c) suggests that milk microbiota is not limited to non-viable bacteria that are associated or not to immune cells and invites us to further address this issue using high-throughput culture-dependent methods. Similar issues have been raised concerning other microbiota associated with meconium, placenta, amniotic fluid, and uterus as recently reviewed by Perez-Muñoz et al. (2017). Discrepancies between culture-dependent and culture-independent studies, the low bacterial population, and biases of the molecular methods used question the existence of microbiota within the healthy fetal milieu.

Beyond the questions regarding the evaluation of the viability of milk bacteria, questions can be raised about a potential role of non-viable bacteria for both infant’s and mother’s health, especially on the infant GIT or mother mammary gland immune system, in a similar way to inactivated probiotics that have been shown to interact with host cells (Popović et al., 2019). Regardless of whether bacteria are alive or not, some bacterial antigens are present and can interact with the host immune system as do inactivated vaccines (Vinod et al., 2015).

The low bacterial counts in milk may justify the questioning of their biological significance. Nevertheless, although their total number in milk is low, bifidobacteria play a crucial role in shaping the infant gastrointestinal microbiota (Jost et al., 2012). Whether bacteria present inside the mammary gland or in the teat/nipple or milk can interact with host cells and influence mammary gland health remains to be evaluated. *In vitro* experiments of interactions between bacteria (mainly pathogens) and bovine mammary gland epithelial cells suggest that even very low concentrations of bacteria above the cell monolayer (∼10^5^ colony-forming units/ml) can induce immune response of cells (Roussel et al., 2015).

### Is It the Mere Result of Contamination?

Another argument against the existence of a milk microbiota is that the DNA amplification techniques may amplify even the tiniest amount of contamination. It is in fact necessary to subject the DNA to several rounds of amplification before being able to describe bacteria in healthy milk. In addition, milk sampling carried out according to the NMC guidelines is perfectly suitable for microbial culture, but it might not be “clean” enough for metataxonomic and metagenomic approaches. A recent study by Metzger et al. (2018a) evaluated the influence of the sampling technique and did also evaluate the impact of different bedding types on the milk microbiota. Adding to conventional sampling, the authors collected milk samples directly from the gland cistern by puncture. Unexpectedly, amplification by PCR was even higher in cisternal samples (83%) when compared to composite and conventional samples (45 and 40%, respectively), strongly suggesting that bacteria are indeed inside the mammary gland and do not get in the milk as a result of external post-contamination during conventional sampling. For cisternal milk, however, the overall bacterial community differed among bedding types, leaving the question open about the environmental influence on the milk microbiota, which somehow contrasts with the “resident resilience” hypothesis (Ganda et al., 2017).

### What About Non-bacterial Components of Milk Microbiota and Microbiota-Associated Functions?

Microbiota may comprise bacteria, viruses, fungi, and other microorganisms. Until now, research has mainly focused on the bacterial component of the milk microbiota. However, a plethora of fungal (Iliev et al., 2012) and viral entities (Virgin et al., 2009) might be present in association with bacteria, with relevant physiological and pathological implications for their host. Exploration of fungal communities as well as bifidophages has just started in human milk (Boix-Amorós et al., 2017, 2019; Duranti et al., 2017) and bovine milk (Derakhshani et al., 2018b). Another aspect is linked to the functions exerted by the milk microbiota. Shotgun metagenomic sequencing can give access to the non-bacterial communities as well as to the functional profiling of milk microbiota (Jiménez et al., 2015; Pärnänen et al., 2018; Hoque et al., 2019). In many cases, the expressed proteins and related biological functions may change although the taxonomical profiles undergo relatively minor variation (Huttenhower et al., 2012). The task of investigating protein expression and microbial functions of the milk microbiota by proteomics is hampered by the very high abundance of host proteins in the milk compared to the microbial counterpart. In addition, sequence information is not available for most of the milk microbiota, creating further difficulties in terms of database annotation and protein identification (Heyer et al., 2017). Looking at the milk metabolome might also provide useful information on microbial functions. However, such approaches are still challenging even in well-characterized microbiota such as in the human GIT (Smirnov et al., 2016).

### How Does Host Genetics Influence the Milk Microbiota?

Several host and environmental factors were reported to influence milk microbiota such as the anatomical characteristics of the teat, farm environment, and housing and management practices including milking practices (Derakhshani et al., 2018a). Few studies have also addressed the impact of host genetics on the bovine milk microbiota. Curone et al. (2018) compared several features of milk from two breeds, the cosmopolitan, highly productive Holstein Friesian and the autochthonous, more rustic Rendena. The study had the advantage of examining two different breeds kept in the same farm and under the same environmental conditions and diets. As a result, Rendena cows showed a lower biodiversity in their milk microbiota than did Holstein and a higher prevalence of *Streptococcus thermophilus*, a thermophilic lactic acid bacterium with a prominent role in dairy product fermentation (Quigley et al., 2013) and with a potential role in protection against mammary pathogens (Nader-Macias et al., 2008; Espeche et al., 2012; Rigobelo et al., 2015). Further characterization of the microbiota composition of 117 healthy quarter milk samples of the two breeds during their periparturient period showed a significantly lower bacterial biodiversity and a more stable microbiota in Rendena milk, while Holstein milk displayed more significant changes in their milk bacterial composition (Cremonesi et al., 2018). Interesting differences in the predicted functional profiles were also detected. The author findings suggest that breed, and therefore host genetics, may have an influence on the milk microbiota composition, with consequences on dairy product characteristics but also on susceptibility to disease and resistance to bacterial infection. In a preliminary, small-scale, genome-wide association study, Huson et al. (2018) were able to identify regions in the Holstein cow genome that were potentially associated with the microbial profiles of milk. In a more targeted approach, Derakhshani et al. (2018b) reported a relation between the bovine major histocompatibility complex (BoLA) gene polymorphism and colostrum microbial composition.

### Does the Milk Microbiota Have an Influence on the Calf Gastrointestinal Microbiota?

As outlined above, a role for the milk microbiota in seeding the infant microbiota has been suggested by several studies carried out in women (Matsumiya et al., 2002; Martín et al., 2003; Gueimonde et al., 2007; Makino et al., 2011; Rautava et al., 2012; Fernández et al., 2013). Studies of vertical transfer of microorganisms from ruminant milk to the offspring are scarce. Indeed, it is common practice to separate dairy cow and calf shortly after birth. In a recent study investigating the relationships between maternal microbiota and the early successional development of the calf GIT microbiome, the colostrum microbiota was found to share approximately 10.6 and 9.6% of OTUs with luminal and mucosal microbiota of the calves, respectively, suggesting colostrum contributed to the makeup of the calf GIT (Yeoman et al., 2018). If a role for the milk microbiota in the maturation of the calf gastrointestinal microbiota is demonstrated, we might need to re-evaluate the current calf management strategies in terms of removal from the dam, colostrum and milk pasteurization, or feeding milk replacer and waste milk to calves. Clearly, far more research will be required for understanding this correlation, also when considering the different physiology of the bovine mammary gland and digestive tract when compared to humans.

### Does Antibiotherapy Influence the Milk Microbiota Resistome?

Routine cow management involves the administration of intramammary antibiotics to cows at the beginning of the dry period, aimed at preventing the establishment or persistence of intramammary infection at the following lactation. The standard practice has long been to treat all quarters from all cows (blanket approach) (Smith et al., 1967). Several countries have now opted for treatment only of quarters with previous risk for mastitis in the new lactation (e.g. high somatic cell count or positivity to culture) (Scherpenzeel et al., 2016). Concerns have been raised for the possible impact of the blanket approach on the selection of antibiotic resistance bacteria (Oliver and Murinda, 2012). In addition, intramammary antibiotics administered to healthy quarters may have an influence on the microbiota and increase the pool of antimicrobial resistance genes. The availability of culture-independent metagenomic methods that target genetic material recovered directly from samples will be of help in understanding the role of non-cultivable bacteria and in characterizing the resistome (Perry et al., 2014). In line with this major concern, a functional profiling of bovine milk microbiota has notably revealed several metabolic pathways related to antibiotic and heavy metal resistance, which likely resulted from a wide use of these compounds (Hoque et al., 2019). The human milk resistome has also started to be explored, suggesting a role in shaping infant gastrointestinal resistome (Pärnänen et al., 2018). Additional functional traits that have been shown to contribute to antibiotic resistance should also be examined when considering the resistome, such as the ability to form biofilms (Hall and Mah, 2017).

### Can We Manipulate the Milk Microbiota in Order to Improve Mammary Gland or Offspring Health?

Studies in women suggest the existence of an enteromammary route of microbial transfer, with possible applications of maternal probiotic supplementation for modulating the offspring gastrointestinal microbiota as well as mammary gland health. Therefore, events that alter the gastrointestinal microbiota might affect the milk microbiota as well. This was investigated in human milk microbiota for antibiotic treatment, which was shown to affect the content in bifidobacteria and lactobacilli (Soto et al., 2014). The human milk microbiota was also found to be related to nutrient intake including fatty acid, carbohydrate, or protein intakes (Williams et al., 2017).

Studies in women support the protective role of the mammary gland microbiota against infection. Arroyo et al. (2010) showed that oral administration of lactobacilli isolated from the milk of healthy women was more effective than antibiotic therapy in treating mastitis. Moreover, bacteria in treated women who had no lactobacilli in their milk before treatment were colonized by the lactobacilli strains used in the trial, suggesting that probiotic mastitis treatment approaches may be feasible (Arroyo et al., 2010).

On the other hand, in a very recent study on women, Simpson et al. (2018) investigated the effect of maternal supplementation with *Lactobacillus rhamnosus* GG, *Lactobacillus acidophilus* La-5, and *Bifidobacterium animalis* ssp. *lactis* Bb-12 on the milk microbiota and on the infant gastrointestinal microbiota. As a result, they concluded that breastfeeding was unlikely to be a significant source of these probiotics for infants. In addition, oral administration to the mothers did not significantly affect the overall composition of the milk microbiota.

In bovines, intramammary infusion with lactococci has been proposed to be as effective at eliminating chronic subclinical infections as an antibiotic treatment (Klostermann et al., 2008). However, rather than reconstituting the microbiota equilibrium, some authors argue that this might be due to an immune stimulation enabling a response of the mammary gland and clearance of subclinical intramammary infection agents (Crispie et al., 2008; Mignacca et al., 2017). In line with this concept, Pellegrino et al. (2017) described that inoculation of lactic acid bacteria in cows at dry-off period increased the amount of IgG isotypes in blood and milk and found that these antibodies were able to recognize *S. aureus* epitopes. Lymphocyte proliferation was significantly higher in the inoculated group at all time points assayed, following lactic acid bacteria or *S. aureus* stimulation. The results showed that probiotics could be a natural and effective alternative in the prevention of bovine mastitis at dry-off period and act as an immunomodulator stimulating local and systemic defense lines (Pellegrino et al., 2017). On the other hand, other studies suggest that intramammary probiotics should be considered with caution, although teat apex probiotics deserve further research (Rainard and Foucras, 2018). Clearly, more research is needed for understanding the impact of administration of probiotics on the cow milk microbiota as well as on the offspring gastrointestinal microbiota and on their respective health status.

## Conclusion

The discovery of an unsuspected complex microbiota associated with milk isolated from healthy hosts has considerably changed our perception of this essential “fluid.” The existence of milk microbiota has been largely substantiated, in both human and cows, revealing a diversity higher than previously suspected. Milk microbiota composition has been shown to be related to several host and environmental factors. By using a cross-species approach, this review allowed us to draw more robust and universal conclusions. Despite a high variability between studies, due to these factors as well as to some technical biases, comparison of milk microbiota in different hosts points out that several taxa, which are among dominant or most frequently cited taxa, are shared between human and animals, inviting us to consider the existence of a core milk microbiota. Whether the existence of interspecies core milk microbiota makes sense and implies a specific role of its members remains to be determined. Nevertheless, this review clearly points out some limitations of the past and present studies on milk microbiota. Much more research is needed on the non-bacterial fraction of this microbiota (i.e. archaeal, fungal, and viral communities) and the interaction network between these different communities as well as on the functional profiling of this microbiota. Many questions need to be answered on viability, origin, and factors driving its composition, before we can implement our findings on the milk microbiota in strategies to address mother and infant health issues or in dairy cow management practice. The potential role of milk and milk-associated microbiota in both infant’s and mother’s health has just started to be explored, supporting a role of milk microbiota in infant gastrointestinal colonization and suggesting a relation between mother’s health and milk microbiota composition. Deciphering factors that shape milk microbiota and relations between milk microbiota and health at short and long terms will open avenues to new strategies for human and animal health management.

## Author Contributions

All authors contributed equally to this manuscript and read and approved the final manuscript. SE and YL conceptualized this review and compiled and harmonized the different contributions.

## Conflict of Interest

The authors declare that the research was conducted in the absence of any commercial or financial relationships that could be construed as a potential conflict of interest.

## Abbreviations

DCT: dry cow therapy
TTGE: temporal temperature gel electrophoresis.

## References

[B1] Aakko J. Kumar H. Rautava S. Wise A. Autran C. Bode L. (2017). Human milk oligosaccharide categories define the microbiota composition in human colostrum. *Benef. Microbes* 8 563–567. 10.3920/BM2016.0185 28726512

[B2] Addis M. F. Tanca A. Uzzau S. Oikonomou G. Bicalho R. C. Moroni P. (2016). The bovine milk microbiota: insights and perspectives from -omics studies. *Mol. Biosyst.* 12 2359–2372. 10.1039/C6MB00217J 27216801

[B3] Arroyo R. Martin V. Maldonado A. Jimenez E. Fernandez L. Rodriguez J. M. (2010). Treatment of infectious mastitis during lactation: antibiotics versus oral administration of Lactobacilli isolated from breast milk. *Clin. Infect. Dis.* 50 1551–1558. 10.1086/652763 20455694

[B4] Avershina E. Angell I. L. Simpson M. Storrø O. Øien T. Johnsen R. (2018). Low maternal microbiota sharing across gut, breast milk and vagina, as revealed by 16S rRNA gene and reduced metagenomic sequencing. *Genes* 9:231. 10.3390/genes9050231 29724017 PMC5977171

[B5] Barthel A. Gourinat A.-C. Cazorla C. Joubert C. Dupont-Rouzeyrol M. Descloux E. (2013). Breast milk as a possible route of vertical transmission of dengue virus? *Clin. Infect. Dis.* 57 415–417. 10.1093/cid/cit227 23575200

[B6] Bhatt V. D. Ahir V. B. Koringa P. G. Jakhesara S. J. Rank D. N. Nauriyal D. S. (2012). Milk microbiome signatures of subclinical mastitis-affected cattle analysed by shotgun sequencing. *J. Appl. Microbiol.* 112 639–650. 10.1111/j.1365-2672.2012.05244.x 22277077

[B7] Biagi E. Quercia S. Aceti A. Beghetti I. Rampelli S. Turroni S. (2017). The bacterial ecosystem of mother’s milk and infant’s mouth and gut. *Front. Microbiol.* 8:1214 10.3389/fmicb.2017.01214 PMC5491547 28713343

[B8] Boix-Amoròs A. Collado M. C. Mira A. (2016). Relationship between milk microbiota, bacterial load, macronutrients and human cells during lactation. *Front. Microbiol.* 7:492 10.3389/fmicb.2016.00492 PMC4837678 27148183

[B9] Boix-Amorós A. Martinez-Costa C. Querol A. Collado M. C. Mira A. (2017). Multiple approaches detect the presence of fungi in human breastmilk samples from healthy mothers. *Sci. Rep.* 7:13016. 10.1038/s41598-017-13270-x 29026146 PMC5638952

[B10] Boix-Amorós A. Puente-Sánchez F. du Toit E. Linderborg K. M. Zhang Y. Yang B. (2019). Mycobiome profiles in breast milk from healthy women depend on mode of delivery, geographic location, and interaction with bacteria. *Appl. Environ. Microbiol.* 85:e02994-18. 10.1128/AEM.02994-18 30824446 PMC6495746

[B11] Bonsaglia E. C. R. Gomes M. S. Canisso I. F. Zhou Z. Lima S. F. Rall V. L. M. (2017). Milk microbiome and bacterial load following dry cow therapy without antibiotics in dairy cows with healthy mammary gland. *Sci. Rep.* 7:8067. 10.1038/s41598-017-08790-5 28808353 PMC5556035

[B12] Borghi E. Massa V. Severgnini M. Fazio G. Avagliano L. Menegola E. (2018). Antenatal microbial colonization of mammalian gut. *Reprod. Sci.* 26 1045–1053. 10.1177/1933719118804411 30309297 PMC6661723

[B13] Bradley A. J. Green M. J. (2004). The importance of the nonlactating period in the epidemiology of intramammary infection and strategies for prevention. *Vet. Clin. North Am. Food Anim. Pract.* 20 547–568. 10.1016/j.cvfa.2004.06.010 15471624

[B14] Braem G. De Vliegher S. Verbist B. Heyndrickx M. Leroy F. De Vuyst L. (2012). Culture-independent exploration of the teat apex microbiota of dairy cows reveals a wide bacterial species diversity. *Vet. Microbiol.* 157 383–390. 10.1016/j.vetmic.2011.12.031 22266158

[B15] Cabrera-Rubio R. Collado M. C. Laitinen K. Salminen S. Isolauri E. Mira A. (2012). The human milk microbiome changes over lactation and is shaped by maternal weight and mode of delivery. *Am. J. Clin. Nutr.* 96 544–551. 10.3945/ajcn.112.037382 22836031

[B16] Castelino M. Eyre S. Moat J. Fox G. Martin P. Ho P. (2017). Optimisation of methods for bacterial skin microbiome investigation: primer selection and comparison of the 454 versus MiSeq platform. *BMC Microbiol.* 17:23. 10.1186/s12866-017-0927-4 28109256 PMC5251215

[B17] Castro S. L. Nelman-Gonzalez M. Nickerson C. A. Ott C. M. (2011). Induction of attachment-independent biofilm formation and repression of Hfq expression by low-fluid-shear culture of *Staphylococcus aureus*. *Appl. Environ. Microbiol.* 77 6368–6378. 10.1128/AEM.00175-11 21803898 PMC3187170

[B18] Catozzi C. Sanchez Bonastre A. Francino O. Lecchi C. De Carlo E. Vecchio D. (2017). The microbiota of water buffalo milk during mastitis. *PLoS One* 12:e0184710. 10.1371/journal.pone.0184710 28926595 PMC5604978

[B19] Cerdó T. Ruiz A. Acuña I. Jáuregui R. Jehmlich N. Haange S.-B. (2018). Gut microbial functional maturation and succession during human early life. *Environ. Microbiol.* 20 2160–2177. 10.1111/1462-2920.14235 29687552

[B20] Chaves Lopez C. Serio A. Rossi C. Mazzarrino G. Marchetti S. Castellani F. (2016). Effect of diet supplementation with *Ascophyllum nodosum* on cow milk composition and microbiota. *J. Dairy Sci.* 99 6285–6297. 10.3168/jds.2015-10837 27320666

[B21] Cooke F. J. Ginwalla S. Hampton M. D. Wain J. Ross-Russell R. Lever A. (2009). Report of neonatal meningitis due to *Salmonella enterica* serotype Agona and review of breast milk-associated neonatal *Salmonella* infections. *J. Clin. Microbiol.* 47 3045–3049. 10.1128/JCM.01064-09 19605582 PMC2738062

[B22] Cremonesi P. Ceccarani C. Curone G. Severgnini M. Pollera C. Bronzo V. (2018). Milk microbiome diversity and bacterial group prevalence in a comparison between healthy Holstein Friesian and Rendena cows. *PLoS One* 13:e0205054. 10.1371/journal.pone.0205054 30356246 PMC6200206

[B23] Crispie F. Alonso-Gomez M. O’Loughlin C. Klostermann K. Flynn J. Arkins S. (2008). Intramammary infusion of a live culture for treatment of bovine mastitis: effect of live lactococci on the mammary immune response. *J. Dairy Res.* 75 374–384. 10.1017/S0022029908003385 18680623

[B24] Curone G. Filipe J. Cremonesi P. Trevisi E. Amadori M. Pollera C. (2018). What we have lost: mastitis resistance in Holstein Friesians and in a local cattle breed. *Res. Vet. Sci.* 116 88–98. 10.1016/j.rvsc.2017.11.020 29223308

[B25] Davé V. Street K. Francis S. Bradman A. Riley L. Eskenazi B. (2016). Bacterial microbiome of breast milk and child saliva from low-income Mexican-American women and children. *Pediatr. Res.* 79 846–854. 10.1038/pr.2016.9 26756784 PMC4899194

[B26] Derakhshani H. Fehr K. B. Sepehri S. Francoz D. De Buck J. Barkema H. W. (2018a). Invited review: microbiota of the bovine udder: contributing factors and potential implications for udder health and mastitis susceptibility. *J. Dairy Sci.* 101 10605–10625. 10.3168/jds.2018-14860 30292553

[B27] Derakhshani H. Plaizier J. C. De Buck J. Barkema H. W. Khafipour E. (2018b). Association of bovine major histocompatibility complex (BoLA) gene polymorphism with colostrum and milk microbiota of dairy cows during the first week of lactation. *Microbiome* 6:203. 10.1186/s40168-018-0586-1 30419937 PMC6233267

[B28] Derakhshani H. Plaizier J. C. De Buck J. Barkema H. W. Khafipour E. (2018c). Composition of the teat canal and intramammary microbiota of dairy cows subjected to antimicrobial dry cow therapy and internal teat sealant. *J. Dairy Sci.* 101 10191–10205. 10.3168/jds.2018-14858 30172408

[B29] Dolci P. De Filippis F. La Storia A. Ercolini D. Cocolin L. (2014). rRNA-based monitoring of the microbiota involved in Fontina PDO cheese production in relation to different stages of cow lactation. *Int. J. Food Microbiol.* 185 127–135. 10.1016/j.ijfoodmicro.2014.05.021 24960294

[B30] Donnet-Hughes A. Perez P. F. Doré J. Leclerc M. Levenez F. Benyacoub J. (2010). Potential role of the intestinal microbiota of the mother in neonatal immune education. *Proc. Nutr. Soc.* 69 407–415. 10.1017/S0029665110001898 20633308

[B31] Doyle C. J. Gleeson D. O’Toole P. W. Cotter P. D. (2017). Impacts of seasonal housing and teat preparation on raw milk microbiota: a high-throughput sequencing study. *Appl. Environ. Microbiol.* 83:e02694-16. 10.1128/AEM.02694-16 27815277 PMC5203630

[B32] Duranti S. Lugli G. A. Mancabelli L. Armanini F. Turroni F. James K. (2017). Maternal inheritance of bifidobacterial communities and bifidophages in infants through vertical transmission. *Microbiome* 5:66. 10.1186/s40168-017-0282-6 28651630 PMC5485682

[B33] Erkus O. de Jager V. C. L. Geene R. T. C. M. van Alen-Boerrigter I. Hazelwood L. van Hijum S. A. F. T. (2016). Use of propidium monoazide for selective profiling of viable microbial cells during Gouda cheese ripening. *Int. J. Food Microbiol.* 228 1–9. 10.1016/j.ijfoodmicro.2016.03.027 27077825

[B34] Escudié F. Auer L. Bernard M. Mariadassou M. Cauquil L. Vidal K. (2018). FROGS: find, rapidly, OTUs with galaxy solution. *Bioinformatics* 34 1287–1294. 10.1093/bioinformatics/btx791 29228191

[B35] Espeche M. C. Pellegrino M. Frola I. Larriestra A. Bogni C. Nader-Macias M. E. (2012). Lactic acid bacteria from raw milk as potentially beneficial strains to prevent bovine mastitis. *Anaerobe* 18 103–109. 10.1016/j.anaerobe.2012.01.002 22261519

[B36] Esteban-Blanco C. Gutiérrez-Gil B. Puente-Sánchez F. Marina H. Tamames J. Acedo A. (2019). Microbiota characterization of sheep milk and its association with somatic cell count using 16s rRNA gene sequencing. *J. Anim. Breed. Genet.* 137 73–83. 10.1111/jbg.12446 31602717

[B37] Falentin H. Rault L. Nicolas A. Bouchard D. S. Lassalas J. Lamberton P. (2016). Bovine teat microbiome analysis revealed reduced alpha diversity and significant changes in taxonomic profiles in quarters with a history of mastitis. *Front. Microbiol.* 7:480. 10.3389/fmicb.2016.00480 27242672 PMC4876361

[B38] Fernández L. Langa S. Martín V. Maldonado A. Jiménez E. Martín R. (2013). The human milk microbiota: origin and potential roles in health and disease. *Pharmacol. Res.* 69 1–10. 10.1016/j.phrs.2012.09.001 22974824

[B39] Fitzstevens J. L. Smith K. C. Hagadorn J. I. Caimano M. J. Matson A. P. Brownell E. A. (2016). Systematic review of the human milk microbiota. *Nutr. Clin. Pract.* 32 354–364. 10.1177/0884533616670150 27679525

[B40] Fouhy F. Clooney A. G. Stanton C. Claesson M. J. Cotter P. D. (2016). 16S rRNA gene sequencing of mock microbial populations- impact of DNA extraction method, primer choice and sequencing platform. *BMC Microbiol.* 16:123. 10.1186/s12866-016-0738-z 27342980 PMC4921037

[B41] Frétin M. Martin B. Rifa E. Isabelle V.-M. Pomiès D. Ferlay A. (2018). Bacterial community assembly from cow teat skin to ripened cheeses is influenced by grazing systems. *Sci. Rep.* 8:200. 10.1038/s41598-017-18447-y 29317671 PMC5760519

[B42] Ganda E. K. Bisinotto R. S. Lima S. F. Kronauer K. Decter D. H. Oikonomou G. (2016). Longitudinal metagenomic profiling of bovine milk to assess the impact of intramammary treatment using a third-generation cephalosporin. *Sci. Rep.* 6:37565. 10.1038/srep37565 27874095 PMC5118806

[B43] Ganda E. K. Gaeta N. Sipka A. Pomeroy B. Oikonomou G. Schukken Y. H. (2017). Normal milk microbiome is reestablished following experimental infection with *Escherichia coli* independent of intramammary antibiotic treatment with a third-generation cephalosporin in bovines. *Microbiome* 5:74. 10.1186/s40168-017-0291-5 28701174 PMC5506599

[B44] Gill J. J. Sabour P. M. Gong J. Yu H. Leslie K. E. Griffiths M. W. (2006). Characterization of bacterial populations recovered from the teat canals of lactating dairy and beef cattle by 16S rRNA gene sequence analysis. *FEMS Microbiol. Ecol.* 56 471–481. 10.1111/j.1574-6941.2006.00091.x 16689878

[B45] González R. Maldonado A. Martín V. Mandomando I. Fumadó V. Metzner K. J. (2013). Breast milk and gut microbiota in African mothers and infants from an area of high HIV prevalence. *PLoS One* 8:e80299. 10.1371/journal.pone.0080299 24303004 PMC3841168

[B46] Gueimonde M. Laitinen K. Salminen S. Isolauri E. (2007). Breast milk: a source of bifidobacteria for infant gut development and maturation? *Neonatology* 92 64–66. 10.1159/000100088 17596738

[B47] Hall C. W. Mah T.-F. (2017). Molecular mechanisms of biofilm-based antibiotic resistance and tolerance in pathogenic bacteria. *FEMS Microbiol. Rev.* 41 276–301. 10.1093/femsre/fux010 28369412

[B48] Hermansson H. Kumar H. Collado M. C. Salminen S. Isolauri E. Rautava S. (2019). Breast milk microbiota is shaped by mode of delivery and intrapartum antibiotic exposure. *Front. Nutr.* 6:4. 10.3389/fnut.2019.00004 30778389 PMC6369203

[B49] Heyer R. Schallert K. Zoun R. Becher B. Saake G. Benndorf D. (2017). Challenges and perspectives of metaproteomic data analysis. *J. Biotechnol.* 261 24–36. 10.1016/j.jbiotec.2017.06.1201 28663049

[B50] Hoque M. N. Istiaq A. Clement R. A. Sultana M. Crandall K. A. Siddiki A. Z. (2019). Metagenomic deep sequencing reveals association of microbiome signature with functional biases in bovine mastitis. *Sci. Rep.* 9:13536. 10.1038/s41598-019-49468-4 31537825 PMC6753130

[B51] Hunt K. M. Foster J. A. Forney L. J. Schutte U. M. E. Beck D. L. Abdo Z. (2011). Characterization of the diversity and temporal stability of bacterial communities in human milk. *PLoS One* 6:e21313. 10.1371/journal.pone.0021313 21695057 PMC3117882

[B52] Huson H. J. Lima S. F. Bicalho R. C. (2018). “16S rRNA milk microbiota profiles on Holstein cows highlight QTL and provide a novel trait to assess the genetic regulation of mastitis,” in *Proceedings of the World Congress on Genetics Applied to Livestock Production Biology & Species-Bovine*, Vol. 2 Belo Horizonte, 508.

[B53] Huttenhower C. Gevers D. Knight R. Abubucker S. Badger J. H. Chinwalla A. T. (2012). Structure, function and diversity of the healthy human microbiome. *Nature* 486 207–214. 10.1038/nature11234 22699609 PMC3564958

[B54] Iliev I. D. Funari V. A. Taylor K. D. Nguyen Q. Reyes C. N. Strom S. P. (2012). Interactions between commensal fungi and the C-type lectin receptor Dectin-1 influence colitis. *Science* 336 1314–1317. 10.1126/science.1221789 22674328 PMC3432565

[B55] Jeurink P. V. van B. J. Jimenez E. Knippels L. M. Fernandez L. Garssen J. (2013). Human milk: a source of more life than we imagine. *Benef. Microbes* 4 17–30. 10.3920/BM2012.0040 23271066

[B56] Jiménez E. de Andrés J. Manrique M. Pareja-Tobes P. Tobes R. Martínez-Blanch J. F. (2015). Metagenomic analysis of milk of healthy and mastitis-suffering women. *J. Hum. Lact.* 31 406–415. 10.1177/0890334415585078 25948578

[B57] Jimenez E. Fernandez L. Maldonado A. Martin R. Olivares M. Xaus J. (2008). Oral administration of *Lactobacillus* strains isolated from breast milk as an alternative for the treatment of infectious mastitis during lactation. *Appl. Environ. Microbiol.* 74 4650–4655. 10.1128/aem.02599-07 18539795 PMC2519365

[B58] Jost T. Lacroix C. Braegger C. Chassard C. (2013). Assessment of bacterial diversity in breast milk using culture-dependent and culture-independent approaches. *Br. J. Nutr.* 110 1253–1262. 10.1017/S0007114513000597 23507238

[B59] Jost T. Lacroix C. Braegger C. Chassard C. (2015). Impact of human milk bacteria and oligosaccharides on neonatal gut microbiota establishment and gut health. *Nutr. Rev.* 73 426–437. 10.1093/nutrit/nuu016 26081453

[B60] Jost T. Lacroix C. Braegger C. P. Chassard C. (2012). New insights in gut microbiota establishment in healthy breast fed neonates. *PLoS One* 7:e44595. 10.1371/journal.pone.0044595 22957008 PMC3431319

[B61] Jost T. Lacroix C. Braegger C. P. Rochat F. Chassard C. (2014). Vertical mother-neonate transfer of maternal gut bacteria via breastfeeding. *Environ. Microbiol.* 16 2891–2904. 10.1111/1462-2920.12238 24033881

[B62] Kang K. P. Lee S. Kang S. K. (2006). D-lactic acidosis in humans: review of update. *Electrolyte Blood Press.* 4 53–56. 10.5049/EBP.2006.4.1.53 24459486 PMC3894545

[B63] Khodayar-Pardo P. Mira-Pascual L. Collado M. C. Martínez-Costa C. (2014). Impact of lactation stage, gestational age and mode of delivery on breast milk microbiota. *J. Perinatol.* 34 599–605. 10.1038/jp.2014.47 24674981

[B64] Kizerwetter-Świda M. Binek M. (2016). Assessment of potentially probiotic properties of *Lactobacillus* strains isolated from chickens. *Pol. J. Vet. Sci.* 19 15–20. 10.1515/pjvs-2016-0003 27096783

[B65] Klindworth A. Pruesse E. Schweer T. Peplies J. Quast C. Horn M. (2013). Evaluation of general 16S ribosomal RNA gene PCR primers for classical and next-generation sequencing-based diversity studies. *Nucleic Acids Res.* 41:e1. 10.1093/nar/gks808 22933715 PMC3592464

[B66] Klostermann K. Crispie F. Flynn J. Ross R. P. Hill C. Meaney W. (2008). Intramammary infusion of a live culture of *Lactococcus lactis* for treatment of bovine mastitis: comparison with antibiotic treatment in field trials. *J. Dairy Res.* 75 365–373. 10.1017/S0022029908003373 18680622

[B67] Knudsen B. E. Bergmark L. Munk P. Lukjancenko O. Priemé A. Aarestrup F. M. (2016). Impact of sample type and DNA isolation procedure on genomic inference of microbiome composition. *mSystems* 1:e00095-16. 27822556 10.1128/mSystems.00095-16 PMC5080404

[B68] Kuehn J. S. Gorden P. J. Munro D. Rong R. Dong Q. Plummer P. J. (2013). Bacterial community profiling of milk samples as a means to understand culture-negative bovine clinical mastitis. *PLoS One* 8:e61959. 10.1371/journal.pone.0061959 23634219 PMC3636265

[B69] Kumar H. du Toit E. Kulkarni A. Aakko J. Linderborg K. M. Zhang Y. (2016). Distinct patterns in human milk microbiota and fatty acid profiles across specific geographic locations. *Front. Microbiol.* 7:1619. 10.3389/fmicb.2016.01619 27790209 PMC5061857

[B70] Lewis Z. T. Totten S. M. Smilowitz J. T. Popovic M. Parker E. Lemay D. G. (2015). Maternal fucosyltransferase 2 status affects the gut bifidobacterial communities of breastfed infants. *Microbiome* 3:13. 10.1186/s40168-015-0071-z 25922665 PMC4412032

[B71] Li Z. Wright A.-D. G. Yang Y. Si H. Li G. (2017). Unique bacteria community composition and co-occurrence in the milk of different ruminants. *Sci. Rep.* 7:40950. 10.1038/srep40950 28098228 PMC5241872

[B72] Lima S. F. Bicalho M. L. de S. Bicalho R. C. (2018). Evaluation of milk sample fractions for characterization of milk microbiota from healthy and clinical mastitis cows. *PLoS One* 13:e0193671. 10.1371/journal.pone.0193671 29561873 PMC5862444

[B73] Lima S. F. Teixeira A. G. V. Lima F. S. Ganda E. K. Higgins C. H. Oikonomou G. (2017). The bovine colostrum microbiome and its association with clinical mastitis. *J. Dairy Sci.* 100 3031–3042. 10.3168/jds.2016-11604 28161185

[B74] Makino H. Kushiro A. Ishikawa E. Muylaert D. Kubota H. Sakai T. (2011). Transmission of intestinal *Bifidobacterium longum* subsp. *longum* strains from mother to infant, determined by multilocus sequencing typing and amplified fragment length polymorphism. *Appl. Environ. Microbiol.* 77 6788–6793. 10.1128/AEM.05346-11 21821739 PMC3187114

[B75] Makovec J. A. Ruegg P. L. (2003). Results of milk samples submitted for microbiological examination in Wisconsin from 1994 to 2001. *J. Dairy Sci.* 86 3466–3472. 10.3168/jds.S0022-0302(03)73951-4 14672176

[B76] Maldonado-Lobón J. A. Díaz-López M. A. Carputo R. Duarte P. Díaz-Ropero M. P. Valero A. D. (2015). *Lactobacillus fermentum* CECT 5716 reduces *Staphylococcus* load in the breastmilk of lactating mothers suffering breast pain: a randomized controlled trial. *Breastfeed. Med.* 10 425–432. 10.1089/bfm.2015.0070 26352805

[B77] Marchesi J. R. Ravel J. (2015). The vocabulary of microbiome research: a proposal. *Microbiome* 3:31. 10.1186/s40168-015-0094-5 26229597 PMC4520061

[B78] Martín R. Jiménez E. Heilig H. Fernández L. Marín M. L. Zoetendal E. G. (2009). Isolation of bifidobacteria from breast milk and assessment of the bifidobacterial population by PCR-denaturing gradient gel electrophoresis and quantitative real-time PCR. *Appl. Environ. Microbiol.* 75 965–969. 10.1128/AEM.02063-08 19088308 PMC2643565

[B79] Martín R. Langa S. Reviriego C. Jimínez E. Marín M. L. Xaus J. (2003). Human milk is a source of lactic acid bacteria for the infant gut. *J. Pediatr.* 143 754–758. 10.1016/j.jpeds.2003.09.028 14657823

[B80] Matsumiya Y. Kato N. Watanabe K. Kato H. (2002). Molecular epidemiological study of vertical transmission of vaginal *Lactobacillus* species from mothers to newborn infants in Japanese, by arbitrarily primed polymerase chain reaction. *J. Infect. Chemother.* 8 43–49. 10.1007/s101560200005 11957119

[B81] McInnis E. A. Kalanetra K. M. Mills D. A. Maga E. A. (2015). Analysis of raw goat milk microbiota: impact of stage of lactation and lysozyme on microbial diversity. *Food Microbiol.* 46 121–131. 10.1016/j.fm.2014.07.021 25475275

[B82] Mediano P. Fernández L. Jiménez E. Arroyo R. Espinosa-Martos I. Rodríguez J. M. (2017). Microbial diversity in milk of women with mastitis: potential role of coagulase-negative staphylococci, viridans group streptococci, and corynebacteria. *J. Hum. Lact.* 33 309–318. 10.1177/0890334417692968 28418794

[B83] Metzger S. A. Hernandez L. L. Skarlupka J. H. Suen G. Walker T. M. Ruegg P. L. (2018a). Influence of sampling technique and bedding type on the milk microbiota: results of a pilot study. *J. Dairy Sci.* 101 6346–6356. 10.3168/jds.2017-14212 29680645

[B84] Metzger S. A. Hernandez L. L. Suen G. Ruegg P. L. (2018b). Understanding the milk microbiota. *Vet. Clin. North Am. Food Anim. Pract.* 34 427–438. 10.1016/j.cvfa.2018.06.003 30316501

[B85] Mignacca S. A. Dore S. Spuria L. Zanghì P. Amato B. Duprè I. (2017). Intramammary infusion of a live culture of *Lactococcus lactis* in ewes to treat staphylococcal mastitis. *J. Med. Microbiol.* 66 1798–1810. 10.1099/jmm.0.000641 29134942

[B86] Moossavi S. Sepehri S. Robertson B. Bode L. Goruk S. Field C. J. (2019). Composition and variation of the human milk microbiota are influenced by maternal and early-life factors. *Cell Host Microbe* 25 324–335.e4. 10.1016/j.chom.2019.01.011 30763539

[B87] Murphy K. Curley D. O’Callaghan T. F. O’Shea C.-A. Dempsey E. M. O’Toole P. W. (2017). The composition of human milk and infant faecal microbiota over the first three months of life: a pilot study. *Sci. Rep.* 7:40597. 10.1038/srep40597 28094284 PMC5240090

[B88] Nader-Macias M. E. Otero M. C. Espeche M. C. Maldonado N. C. (2008). Advances in the design of probiotic products for the prevention of major diseases in dairy cattle. *J. Ind. Microbiol. Biotechnol.* 35 1387–1395. 10.1007/s10295-008-0438-2 18758837

[B89] Nogacka A. M. Salazar N. Arboleya S. Suárez M. Fernández N. Solís G. (2018). Early microbiota, antibiotics and health. *Cell. Mol. Life Sci.* 75 83–91. 10.1007/s00018-017-2670-2 28988290 PMC11105232

[B90] Oikonomou G. Bicalho M. L. Meira E. Rossi R. E. Foditsch C. Machado V. S. (2014). Microbiota of cow’s milk; distinguishing healthy, sub-clinically and clinically diseased quarters. *PLoS One* 9:e85904. 10.1371/journal.pone.0085904 24465777 PMC3896433

[B91] Oikonomou G. Machado V. S. Santisteban C. Schukken Y. H. Bicalho R. C. (2012). Microbial diversity of bovine mastitic milk as described by pyrosequencing of metagenomic 16s rDNA. *PLoS One* 7:e47671. 10.1371/journal.pone.0047671 23082192 PMC3474744

[B92] Olivares M. Albrecht S. De Palma G. Ferrer M. D. Castillejo G. Schols H. A. (2014). Human milk composition differs in healthy mothers and mothers with celiac disease. *Eur. J. Nutr.* 54 119–128. 10.1007/s00394-014-0692-1 24700375

[B93] Oliver S. P. Murinda S. E. (2012). Antimicrobial resistance of mastitis pathogens. *Vet. Clin. North Am. Food Anim. Pract.* 28 165–185. 10.1016/j.cvfa.2012.03.005 22664201

[B94] Pärnänen K. Karkman A. Hultman J. Lyra C. Bengtsson-Palme J. Larsson D. G. J. (2018). Maternal gut and breast milk microbiota affect infant gut antibiotic resistome and mobile genetic elements. *Nat. Commun.* 9:3891. 10.1038/s41467-018-06393-w 30250208 PMC6155145

[B95] Patel S. H. Vaidya Y. H. Patel R. J. Pandit R. J. Joshi C. G. Kunjadiya A. P. (2017). Culture independent assessment of human milk microbial community in lactational mastitis. *Sci. Rep.* 7:7804. 10.1038/s41598-017-08451-7 28798374 PMC5552812

[B96] Pellegrino M. Berardo N. Giraudo J. Nader-Macías M. E. F. Bogni C. (2017). Bovine mastitis prevention: humoral and cellular response of dairy cows inoculated with lactic acid bacteria at the dry-off period. *Benef. Microbes* 8 589–596. 10.3920/BM2016.0194 28701082

[B97] Perez P. F. Doré J. Leclerc M. Levenez F. Benyacoub J. Serrant P. (2007). Bacterial imprinting of the neonatal immune system: lessons from maternal cells? *Pediatrics* 119 e724–e732. 10.1542/peds.2006-1649 17332189

[B98] Perez-Muñoz M. E. Arrieta M.-C. Ramer-Tait A. E. Walter J. (2017). A critical assessment of the “sterile womb” and “in utero colonization” hypotheses: implications for research on the pioneer infant microbiome. *Microbiome* 5:48. 10.1186/s40168-017-0268-4 28454555 PMC5410102

[B99] Perry J. A. Westman E. L. Wright G. D. (2014). The antibiotic resistome: what’s new? *Curr. Opin. Microbiol.* 21 45–50. 10.1016/j.mib.2014.09.002 25280222

[B100] Pham V. T. Lacroix C. Braegger C. P. Chassard C. (2016). Early colonization of functional groups of microbes in the infant gut. *Environ. Microbiol.* 18 2246–2258. 10.1111/1462-2920.13316 27059115

[B101] Pham V. T. Lacroix C. Braegger C. P. Chassard C. (2017). Lactate-utilizing community is associated with gut microbiota dysbiosis in colicky infants. *Sci. Rep.* 7:11176. 10.1038/s41598-017-11509-1 28894218 PMC5593888

[B102] Pollock J. Glendinning L. Wisedchanwet T. Watson M. (2018). The madness of microbiome: attempting to find consensus “best practice” for 16S microbiome studies. *Appl. Environ. Microbiol.* 84:e02627-17. 10.1128/AEM.02627-17 29427429 PMC5861821

[B103] Popović N. Djokić J. Brdarić E. Dinić M. Terzić-Vidojević A. Golić N. (2019). The influence of heat-killed *Enterococcus faecium* BGPAS1-3 on the tight junction protein expression and immune function in differentiated Caco-2 cells infected with *Listeria monocytogenes* ATCC 19111. *Front. Microbiol.* 10:412. 10.3389/fmicb.2019.00412 30891021 PMC6411766

[B104] Quigley L. O’Sullivan O. Stanton C. Beresford T. P. Ross R. P. Fitzgerald G. F. (2013). The complex microbiota of raw milk. *FEMS Microbiol. Rev.* 37 664–698. 10.1111/1574-6976.12030 23808865

[B105] Qutaishat S. S. Stemper M. E. Spencer S. K. Borchardt M. A. Opitz J. C. Monson T. A. (2003). Transmission of *Salmonella enterica* serotype typhimurium DT104 to infants through mother’s breast milk. *Pediatrics* 111 1442–1446. 10.1542/peds.111.6.1442 12777569

[B106] Rainard P. (2017). Mammary microbiota of dairy ruminants: fact or fiction? *Vet. Res.* 48:25. 10.1186/s13567-017-0429-2 28412972 PMC5392980

[B107] Rainard P. Foucras G. (2018). A critical appraisal of probiotics for mastitis control. *Front. Vet. Sci.* 5:251. 10.3389/fvets.2018.00251 30364110 PMC6191464

[B108] Ramsay D. T. Kent J. C. Owens R. A. Hartmann P. E. (2004). Ultrasound imaging of milk ejection in the breast of lactating women. *Pediatrics* 113 361–367. 10.1542/peds.113.2.361 14754950

[B109] Rautava S. (2016). Early microbial contact, the breast milk microbiome and child health. *J. Dev. Orig. Health Dis.* 7 5–14. 10.1017/S2040174415001233 26051698

[B110] Rautava S. Luoto R. Salminen S. Isolauri E. (2012). Microbial contact during pregnancy, intestinal colonization and human disease. *Nat. Rev. Gastroenterol. Hepatol.* 9 565–576. 10.1038/nrgastro.2012.144 22890113

[B111] Rigobelo E. E. C. Karapetkov N. Maestá S. A. Ávila F. A. McIntosh D. (2015). Use of probiotics to reduce faecal shedding of Shiga toxin-producing *Escherichia coli* in sheep. *Benef. Microbes* 6 53–60. 10.3920/BM2013.0094 25380795

[B112] Rintala A. Pietilä S. Munukka E. Eerola E. Pursiheimo J.-P. Laiho A. (2017). Gut microbiota analysis results are highly dependent on the 16S rRNA gene target region, whereas the impact of DNA extraction is minor. *J. Biomol. Tech.* 28 19–30. 10.7171/jbt.17-2801-003 28260999 PMC5330390

[B113] Roussel P. Cunha P. Porcherie A. Petzl W. Gilbert F. B. Riollet C. (2015). Investigating the contribution of IL-17A and IL-17F to the host response during *Escherichia coli* mastitis. *Vet. Res.* 46:56. 10.1186/s13567-015-0201-4 26062913 PMC4462179

[B114] Scherpenzeel C. G. M. den Uijl I. E. M. van Schaik G. Riekerink R. G. M. O. Hogeveen H. Lam T. J. G. M. (2016). Effect of different scenarios for selective dry-cow therapy on udder health, antimicrobial usage, and economics. *J. Dairy Sci.* 99 3753–3764. 10.3168/jds.2015-9963 26947289

[B115] Schwarz D. G. G. Shoyama F. M. Oliveira L. L. Sreevatsan S. Moreira M. A. S. (2018). Rapid baso-apical translocation of *Mycobacterium avium* ssp. *paratuberculosis* in mammary epithelial cells in the presence of *Escherichia coli*. *J. Dairy Sci.* 101 6287–6295. 10.3168/jds.2017-13945 29705415

[B116] Simpson M. R. Avershina E. Storrø O. Johnsen R. Rudi K. Øien T. (2018). Breastfeeding-associated microbiota in human milk following supplementation with *Lactobacillus rhamnosus* GG, *Lactobacillus acidophilus* La-5, and *Bifidobacterium animalis* ssp. *lactis* Bb-12. *J. Dairy Sci.* 101 889–899. 10.3168/jds.2017-13411 29248229

[B117] Smirnov K. S. Maier T. V. Walker A. Heinzmann S. S. Forcisi S. Martinez I. (2016). Challenges of metabolomics in human gut microbiota research. *Int. J. Med. Microbiol.* 306 266–279. 10.1016/j.ijmm.2016.03.006 27012595

[B118] Smith A. Westgarth D. R. Jones M. R. Neave F. K. Dodd F. H. Brander G. C. (1967). Methods of reducing the incidence of udder infection in dry cows. *Vet. Rec.* 81 504–510. 10.1136/vr.81.20.504 5624737

[B119] Soto A. Martín V. Jiménez E. Mader I. Rodríguez J. M. Fernández L. (2014). Lactobacilli and bifidobacteria in human breast milk: influence of antibiotherapy and other host and clinical factors. *J. Pediatr. Gastroenterol. Nutr.* 59 78–88. 10.1097/MPG.0000000000000347 24590211 PMC4086764

[B120] Soto Del Rio M. L. D. Dalmasso A. Civera T. Bottero M. T. (2017). Characterization of bacterial communities of donkey milk by high-throughput sequencing. *Int. J. Food Microbiol.* 251 67–72. 10.1016/j.ijfoodmicro.2017.03.023 28431310

[B121] Toscano M. De Grandi R. Peroni D. G. Grossi E. Facchin V. Comberiati P. (2017). Impact of delivery mode on the colostrum microbiota composition. *BMC Microbiol.* 17:205. 10.1186/s12866-017-1109-0 28946864 PMC5613475

[B122] Tremblay J. Singh K. Fern A. Kirton E. S. He S. Woyke T. (2015). Primer and platform effects on 16S rRNA tag sequencing. *Front. Microbiol.* 6:771. 10.3389/fmicb.2015.00771 26300854 PMC4523815

[B123] Treven P. Mrak V. Bogovič Matijašić B. Horvat S. Rogelj I. (2015). Administration of probiotics *Lactobacillus rhamnosus* GG and *Lactobacillus gasseri* K7 during pregnancy and lactation changes mouse mesenteric lymph nodes and mammary gland microbiota. *J. Dairy Sci.* 98 2114–2128. 10.3168/jds.2014-8519 25622869

[B124] Urbaniak C. Angelini M. Gloor G. B. Reid G. (2016). Human milk microbiota profiles in relation to birthing method, gestation and infant gender. *Microbiome* 4:1. 10.1186/s40168-015-0145-y 26739322 PMC4702315

[B125] Urbaniak C. Cummins J. Brackstone M. Macklaim J. M. Gloor G. B. Baban C. K. (2014). Microbiota of human breast tissue. *Appl. Environ. Microbiol.* 80 3007–3014. 10.1128/AEM.00242-14 24610844 PMC4018903

[B126] Verdier-Metz I. Gagne G. Bornes S. Monsallier F. Veisseire P. Delbès-Paus C. (2012). Cow teat skin, a potential source of diverse microbial populations for cheese production. *Appl. Environ. Microbiol.* 78 326–333. 10.1128/AEM.06229-11 22081572 PMC3255753

[B127] Vilca L. M. Bartolomé R. de Arquer M. Albero I. Ribes C. Campins-Martí M. (2015). Mother as a vector of *Salmonella enterica* serotype Newport outbreak in a neonatal unit. *Enferm. Infecc. Microbiol. Clin.* 33 536–538. 10.1016/j.eimc.2014.10.012 25600024

[B128] Vinod N. Oh S. Park H. J. Koo J. M. Choi C. W. Kim S. C. (2015). Generation of a novel *Staphylococcus aureus* ghost vaccine and examination of its immunogenicity against virulent challenge in rats. *Infect. Immun.* 83 2957–2965. 10.1128/IAI.00009-15 25964469 PMC4468543

[B129] Virgin H. W. Wherry E. J. Ahmed R. (2009). Redefining chronic viral infection. *Cell* 138 30–50. 10.1016/j.cell.2009.06.036 19596234

[B130] Wang L.-Y. Chen C.-T. Liu W.-H. Wang Y.-H. (2007). Recurrent neonatal group B streptococcal disease associated with infected breast milk. *Clin. Pediatr.* 46 547–549. 10.1177/0009922807299467 17579109

[B131] Williams J. E. Carrothers J. M. Lackey K. A. Beatty N. F. Brooker S. L. Peterson H. K. (2019). Strong multivariate relations exist among milk, oral, and fecal microbiomes in mother-infant dyads during the first six months postpartum. *J. Nutr.* 149 902–914. 10.1093/jn/nxy299 31063198 PMC6543206

[B132] Williams J. E. Carrothers J. M. Lackey K. A. Beatty N. F. York M. A. Brooker S. L. (2017). Human milk microbial community structure is relatively stable and related to variations in macronutrient and micronutrient intakes in healthy lactating women. *J. Nutr.* 147 1739–1748. 10.3945/jn.117.248864 28724659 PMC5572491

[B133] Yeoman C. J. Ishaq S. L. Bichi E. Olivo S. K. Lowe J. Aldridge B. M. (2018). Biogeographical differences in the influence of maternal microbial sources on the early successional development of the bovine neonatal gastrointestinal tract. *Sci. Rep.* 8:3197. 10.1038/s41598-018-21440-8 29453364 PMC5816665

[B134] Young W. Hine B. C. Wallace O. A. M. Callaghan M. Bibiloni R. (2015). Transfer of intestinal bacterial components to mammary secretions in the cow. *PeerJ* 3:e888. 10.7717/peerj.888 25922791 PMC4411484

[B135] Zeineldin M. Lowe J. de Godoy M. Maradiaga N. Ramirez C. Ghanem M. (2017). Disparity in the nasopharyngeal microbiota between healthy cattle on feed, at entry processing and with respiratory disease. *Vet. Microbiol.* 208 30–37. 10.1016/j.vetmic.2017.07.006 28888646

[B136] Zeineldin M. M. Lowe J. F. Grimmer E. D. de Godoy M. R. C. Ghanem M. M. Abd El-Raof Y. M. (2017). Relationship between nasopharyngeal and bronchoalveolar microbial communities in clinically healthy feedlot cattle. *BMC Microbiol.* 17:138. 10.1186/s12866-017-1042-2 28645257 PMC5481913

[B137] Zhang R. Huo W. Zhu W. Mao S. (2015). Characterization of bacterial community of raw milk from dairy cows during subacute ruminal acidosis challenge by high-throughput sequencing. *J. Sci. Food Agric.* 95 1072–1079. 10.1002/jsfa.6800 24961605

