# Supplementary material for: Milk Microbiota: What Are We Exactly Talking About?
Source: Front Microbiol. 2020 Feb 14;11:60. doi: 10.3389/fmicb.2020.00060 (PMC7034295; doi:10.3389/fmicb.2020.00060)
Supplement: Supplementary file 1 [file Data_Sheet_1.docx]

**Table S1.** Milk microbiota in different species

| **Host** | **Sample** | **Aim and main result** | **Major taxa** | **Methodology** | **Reference** |
| --- | --- | --- | --- | --- | --- |
| Cow | Teat canal | Bacterial populations recovered from the teat canals of lactating dairy and beef cattle | Dairy samples: *Staphylococcaceae*; Beef samples: *Clostridia* | 16S rRNA clone library | (Gill et al., 2006) |
| Cow | Teat apex | Wide bacterial species diversity on teat apex | *Corynebacterium, Bifidobacterium, Staphylcoccus, Jeotgalicoccus, Aerococcus, Streptococcus, Acinetobacter, Psychrobacter, Enterobacter*, members of *Bacteroidetes* | DGGE (16S rRNA gene V3 region) | (Braem et al., 2012) |
| Cow | Teat skin | Cow teat skin as a potential source of diverse microbial populations for cheese production; breakdown of microbial flow from animal to milk | *Aerococcus, Staphylococcus, Enterococcus, Pediococcus, Pantoea, Enterobacter, Escherichia, Streptococcus, Clavibacter, Arthrobacter gandavensis* | 16S rRNAclone library | (Verdier-Metz et al., 2012) |
| Cow | Milk (quarter milk, composite milk and gland cistern milk) | Influence of sampling technique and bedding type on milk microbiota. First reporting of a microbiome associated to gland cistern milk (collected using a needle and vacuum tube) corresponding to non-mastitis quarters. However, all these gland cistern samples were culture-negative | *Enhydrobacter, Rhodocyclaceae, Janthinobacterium, Prevotella, Succiniclasticum, Xanthomonadaceae, Sediminibacterium, Aerococcaceae, Brevundimonas, Succinivibrionaceae, Burkholderia, Stenotrophomonas, Cupriavidus, Staphylococcus, Bacteroidetes, Corynebacterium* | Illumina  16S rRNA gene V4 region | (Metzger et al., 2018) |
| Cow | Teat skin | Influence of grazing system on cow teat skin, raw milk, and ripened cheeses microbiota; key role of cow teat skin as a reservoir of microbial diversity for raw milk | Teat skin and raw milk: *Corynebacterium, Romboustia, Turicibacter, Dietzia maris.* Milk and cheese: *Lactococcus* and *Lactobacillus* | Illumina  16S rRNA gene V3-V4 region | (Frétin et al., 2018) |
| Donkey | Bulk milk | Variability of donkey bulk milk microbiota, dominated by gram-negative bacteria but including some lactic acid bacteria | *Pseudomonas, Ralstonia, Acinetobacter, Cupriavidus, Citrobacter, Sphingobacterium* | Illumina  16S rRNA gene V4 region | (Soto Del Rio et al., 2017) |

| **Host** | **Sample** | **Aim and main result** | **Major taxa** | **Methodology** | **Reference** |
| --- | --- | --- | --- | --- | --- |
| Goat | Raw milk | Impact of stage of lactation and lysozyme on microbial diversity | *Pseudomonas, Micrococcus, Rhodococcus, Arthrobacter, Stenotrophomonas, Phyllobacterium, Streptococcus, Rhizobium, Staphylococcus, Agrobacterium* | 16S rRNA clone library &Illumina  16S rRNA gene V4 region | (McInnis et al., 2015) |
| Mouse | Mammary gland | Administration of lactic acid probiotics during pregnancy and lactation changes mouse mammary gland microbiota by increasing the proportion of Firmicutes | No clear composition of mouse milk microbiota due to low amount of milk and DNA and a high contamination of samples | Pyrosequencing  16S rRNA gene V5-V6 region | (Treven et al., 2015) |
| Water Deer, Reindeer, Goat | Milk | Significant differences in milk microbiota composition in different ruminants suggesting host microbial adaptation caused by evolution | water deer: *Pseudomonas spp., Acinetobacter spp.*; reindeer: *Hyphomicrobiaceae* family and Halomonas spp.; dairy goat: *Bacillus, Staphylococcus, Pseudomona*s | Illumina MiSeq on 16S rRNA gene V1-V3 region | (Li et al., 2017b) |
| Water Buffalo | Milk | Differential microbial communities and diversity with regard to health status | healthy milk*: Micrococcus, Propionibacterium, 5-7N15, Solibacillus, Staphylococcus, Aerococcus, Facklamia, Trichococcus, Turicibacter, 02d06, SMB53, Clostridium, Acinetobacter, Psychrobacter, Pseudomonas* | Ion Torrent  16S rRNA gene V1-V2 region | (Catozzi et al., 2017) |
| Human | Milk | Milk bacterial communities are more complex than previously reported and often stable over time within an individual (4- week interval) | *Staphylococcus, Streptococcus, Serratia, Pseudomonas, Corynebacterium, Ralstonia, Propionibacterium, Sphingomonas, Bradyrhizobiaceae* | Pyrosequencing 16S rRNA gene V1-V2 region | (Hunt et al., 2011) |
| Human | Milk | Human milk is a major source of bacterial diversity to the neonatal gastrointestinal tract, including gastrointestinalobligate anaerobes | *Staphylococcus, Streptococcus, Propionibacterium, Bifidobacterium, Veillonella, Bacteroides, Faecalibacterium, Roseburia.* | Culture & pyrosequencing 16S rRNA gene V5-V6 region | (Jost et al., 2013) |

| **Host** | **Sample** | **Aim and main result** | **Major taxa** | **Methodology** | **Reference** |
| --- | --- | --- | --- | --- | --- |
| *Cows, sheep, goats and humans* | *Milk* | *The complex microbiota of raw milk: review on microorganisms found in milk and their roles, such as facilitating dairy fermentations (e.g. Lactococcus, Lactobacillus, Streptococcus, Propionibacterium and fungal populations), causing spoilage (e.g. Pseudomonas, Clostridium, Bacillus and other spore-forming or thermoduric microorganisms), promoting health (e.g.* lactobacilli *and* bifidobacteria*) or causing disease (e.g. Listeria, Salmonella, Escherichia coli, Campylobacter and mycotoxin-producing fungi)* | | | (Quigley et al., 2013) |
| *Cow* | *Milk* | *Review on bovine milk microbiota in dairy cows, its role in bovine physiology and health, for the mother and the offspring, factors influencing its composition and consequences on quality of milk and of dairy products* | | | (Addis et al., 2016) |
| *Human* | *Milk* | *Systematic review of the human milk microbiota characterized by culture-independent methods. Points out that Streptococcus and Staphylococcus may be universally predominant in human milk, regardless of differences in geographic location or analytical methods* | | | (Fitzstevens et al., 2016) |

**Table S2.** Origin of milk microbiota

| **Host** | **Sample** | **Aim and main result** | **Major taxa** | **Methodology** | **Reference** |
| --- | --- | --- | --- | --- | --- |
| **Origin of milk microbiota** | | | | | |
| Cow | Milk somatic cells | The presence of few OTUs belonging to *Ruminococcus* and *Bifidobacterium* in milk, blood and feces from the same healthy cow suggests transfer of intestinal bacterial components to mammary secretions | *Staphylococcus, Ruminococcus, Peptostreptococcaceae, Bifidobacterium, Butyrivibrio* | Pyrosequencing 16S rRNA gene V3-V5 region | (Young et al., 2015) |
| Human | Maternal & neonatal faeces, milk | Some gastrointestinal obligate anaerobic genera, including a viable strain of *Bifidobacterium breve*, are shared between maternal faeces, milk and neonatal faeces within one mother–neonate pair | *Staphylococcus, Streptococcus, Pseudomonas, Ralstonia, Bifidobacterium, Rothia, Bacteroides, Propionibacterium, Collinsella, Parabacteroides, Blautia, Alistipes, Clostridium, Coprococcus, Dorea, Faecalibacterium, Oscillobacter, Roseburia, Ruminococcus, Veillonella, Lactobacillus, Enterococcus, Subdoligranulum, Dialister, Escherichia/Shigella, Klebsiella* | Pyrosequencing 16S rRNA gene V5-V6 region & culture (underscored genera) | (Jost et al., 2014) |
| Human | Milk cells, PBMC, feces | Results suggest that intestinally derived bacterial components are transported to the lactating breast within mononuclear cells | *Staphylococcus epidermidis, Streptococcus thermophilus/salivarius, bifidobacterium longum, Bacteroides, Clostridium, Eubacterium, Lactobacillus* | TTGE on 16S rRNA gene V6-V8 region  16S rRNA clone library | (Perez et al., 2007) |
| Human | Milk & infant feces | Presence of the same specific bacterial strains of *Bifidobacterium, Lactobacillus*, and *Staphylococcu*s in human milk and infant feces of mother–child pairs | *Bifidobacterium, Lactobacillus, Staphylococcus* | RAPD, PFGE, MLST | (Martín et al., 2006, 2012) |
| *Human* | *Milk* | *The human milk microbiota: Origin and potential roles in health and disease: different origin for milk microbiota, including breast skin, infant oral microbiota as well as the entero-mammary pathways* | | | (Fernández et al., 2013) |
| *Human* | *Milk* | *Human milk: a complex ecosystem with a greater diversity than previously anticipated, possibly originating from the mother’s skin or infant’s oral cavity (contamination) and or through an active migration of bacteria-containing immune cells from gastrointestinal tract to mammary gland* | | | (Jeurink et al., 2013) |

**Table S3.** Factors affecting milk microbiota

| **Host** | **Sample** | **Aim and main result** | **Major taxa** | **Methodology** | **Reference** |
| --- | --- | --- | --- | --- | --- |
| Cow | Milk from individual cows, teat, and bulk tank milk | Influence of environment and farm management practices (housing, teat preparation) on milk microbiota | Milk from individual cow: *Micrococcaceae, Ruminococcaceae, Bacteroidaceae, Corynebacteriaceae, Propionibacteriaceae, Prevotellaceae, Rikenellaceae, Flavobacteriaceae, Staphylococcaceae, Aerococcaceae, Carnobacteriaceae, Streptococcaceae, Christensenellaceae, Lachnospiraceae, Peptostreptococcaceae, Erysipelotrichaceae, Moraxellaceae, Pseudomonadaceae* | Illumina  16S rRNA gene V3-V4 region | (Doyle et al., 2017) |
| Cow | Milk | Resilience of mammary gland microbiome, capable of reestablishing itself after experimental infection with *E. coli* independent of antimicrobial treatment by Ceftiofur | *Ruminococcaceae, Enterobacteriaceae, Aerococcaceae, Lachnospiraceae, Corynebacteriaceae, Planococcaceae,*  *Bacillacea, Clostridiaceae, Bacteroidaceae,Staphylococcaceae* | Illumina  16s rRNA gene V4 region | (Ganda et al., 2016, 2017) |
| Cow | Milk | Influence of breed on the milk microbiota. Different breeds farmed under the same conditions have a different milk microbiota. Cosmopolitan, highly productive Holstein show a higher biodiversity in terms of bacterial phyla, genera, and species when compared to the minor, autochthonous Rendena | Holstein: *Streptococcus, Lactobacillus, Corynebacterium, Staphylococcus, Bradyrhizobium, Clostridium, Aerococcus, Bacteroides, Blautia, Alkaliphilus, Pediococcus, Bifidobacterium;*  Rendena: *Streptococcus, Lactobacillus, Pediococcus, Staphylococcus, Leuconostoc, Lactococcus, Enterococcus* | Illumina 16s rRNA gene V3-V4 region | (Curone et al., 2018) |

| **Host** | **Sample** | **Aim and main result** | **Major taxa** | **Methodology** | **Reference** |
| --- | --- | --- | --- | --- | --- |
| Cow | Colostrum and milk | Influence of breed and stage of lactation on the milk microbiome. Holstein: increase in *Streptococcus*, *Lactobacillus* and *Bradyrhizobium* near the calving period, followed by a decrease in full lactation. Rendena: slight decrease in *Streptococcus*, *Lactobacillus* and *Pediococcus* right before and after calving but recovery in full lactation | Holstein: *Bradyrhizobium, Corynebacterium, Lactobacillus, Propionibacterium,* SMB53, *Staphylococcus, Streptococcus*;  Rendena: *Enterococcus, Lactobacillus, Lactococcus, Leuconostoc, Pediococcus, Streptococcus* | Illumina 16s rRNA gene V3-V4 region | (Cremonesi et al., 2018) |
| Cow | Teat canal, milk and colostrum | Impact of dry cow therapy (DCT): qualitative overlap between pre-DCT milk samples and colostrum microbiota but change in proportions of several taxa and a lower richness in colostrum. Several mastitis pathogens or opportunists, including Staphylococcus spp., unclassified Enterobacteriaceae, and Corynebacterium spp., were shared between pre-DCT and postpartum microbiota of mammary secretions, suggesting a limited success of the DCT in eliminating potential pathogens | Teat canal: Firmicutes (*Ruminococcaceae, Lachnospiraceae, Clostridiales, Bacillales, Staphylococcus)*, Proteobacteria (*Acinetobacter)*, Bacteroidetes *(Spingobacterium, Flavobacteriaceae),* Actinobacteria (*Corynebacterium*)  Milk/colostrum: Proteobacteria *(Alcaligenaceae, Stenotrophomonas, Acinetobacter, Cellvibrio),* Firmicutes *(Clostridiales, Staphylococcus, Ruminococcaceae, Bacillales, Lachnospiraceae),* Bacteroidetes *(Spingobacterium, Flavobacteriaceae),* Actinobacteria *(Corynebacterium)* | Illumina  16S rRNA gene V1-V2 region | (Derakhshani et al., 2018b) |
| Cow | Raw milk | rRNA-based monitoring of the microbiota involved in Fontina PDO cheese production in relation to different stages of cow lactation | Raw milk before cheese making: *Lactobacillus, Propionibacterium acnes, Staphylococcus, Pseudomonas, Enterobacteriaceae, Acinetobacter, Acidovorax, Hymenobacter, Brochothrix, Actinobacteria, Cyanobacteria* | pyrosequencing 16S rRNA gene V1-V3 region (amplification on cDNA) | (Dolci et al., 2014) |

| **Host** | **Sample** | **Aim and main result** | **Major taxa** | **Methodology** | **Reference** |
| --- | --- | --- | --- | --- | --- |
| Cow | Tanker milk | Despite highly diverse tanker milk community structures, distinct milk bacterial communities are selected within the processing facility environment | *Streptococcus, Staphylococcus* and unidentified members of *Clostridiales, Corynebacterium* | Illumina  16S rRNA gene V4 region | (Kable et al., 2016) |
| Cow | Pooled milk | Effect of diet supplementation with *Ascophyllum nodosum* on cow milk microbiota | *Pseudomonas, Lactococcus, Staphylococcus, Bacteroides, Enterococcus, Clostridium, Microbacterium* | Pyrosequencing 16S rRNA gene V1-V3 region (amplification on cDNA) | (Chaves Lopez et al., 2016) |
| Cow | Pooled milk of one day | Impact of subacute ruminal acidosis (SARA), induced by a high concentrate diet, on milk microbiota: increase of some mastitis causing pathogen abundance | *Chryseobacterium, Corynebacterium, Enterococcus, Microbacterium, Streptococcus* | Pyrosequencing 16S rRNA gene V1-V3 region | (Zhang et al., 2015) |
| Human | Colostrum | Impact of delivery mode on colostrum microbiota composition and microbiota interaction networks | *Staphylococcus, Streptococcus, Prevotella, Halomonas, Finegoldia, Haemophilus, Pseudomonas* | Ion Torrent sequencing on 16S rRNA gene V2-V3 region | (Toscano et al., 2017) |
| Human | Colostrum | Correlation between Human milk oligosaccharide composition and *Bifidobacterium* spp. but also *Akkermansia muciniphila* or *Staphylococcus aureus* | *Bifidobacterium, Staphylococcus, Akkermansia* | Quantitative PCR | (Aakko et al., 2017) |
| Human | Milk at 2d and 6m | Factors affecting human milk microbiome include maternal nutrient intake, maternal body mass index, delivery mode, and infant sex | *Streptococcus, Staphylococcus, Propionibacterium, Pseudomonas, Veillonella, Pilibacter, Gemella, Bacteroides, Prevotella, Corynebacterium* | Illumina  16S rRNA gene | (Williams et al., 2017) |

| **Host** | **Sample** | **Aim and main result** | **Major taxa** | **Methodology** | **Reference** |
| --- | --- | --- | --- | --- | --- |
| Human | Milk | No significant effect of delivery mode, infant sex and gestation time on milk microbiota | *Staphylococcus, Pseudomonas, Streptococcus* and *Lactobacillus* | Illumina  16S rRNA gene V6 region | (Urbaniak et al., 2016) |
| Human | Milk | High variability in microbiota composition and bacterial load among mothers. Bacteria present in milk in a planktonic state, but also associated to human immune cells. No correlation between bacterial load and the amount of immune cells, strengthening the idea that milk bacteria are not sensed as an infection by the immune system | *Staphylococcus, Pseudomonas, Streptococcus, Acinetobacter* | Pyrosequencing 16S rRNA gene | (Boix-Amoròs et al., 2016) |
| Human | Milk | Impact of geographical locations and mode of delivery on milk microbiota and lipid composition | *Staphylococcus, Streptococcus, Pseudomonas, Ralstonia, Acinetobacter* | Illumina  16S rRNA gene V4 region | (Kumar et al., 2016) |
| Human | Colostrum and milk | The human milk microbiome changes over lactation and is shaped by maternal weight (lower diversity in milk from obese mothers) and mode of delivery | Colostrum: *Weisella, Leuconostoc, Staphylococcus, Streptococcus, Lactococcus, Acinetobacter, Citrobacter*  1- and 6-mo milk samples: + *Veillonella, Enterococcus, Leptotrichia, Prevotella* | Pyrosequencing on 16S rRNA gene | (Cabrera-Rubio et al., 2012) |
| Human |  | Impact of geographical region (Taiwan and Mainland China) and mode of delivery on milk microbiota. No significant impact of lactation stage or maternal body mass index | *Streptococcaceae, Pseudomonadaceae Staphylococcaceae, Lactobacillaceae, Oxalobacteraceae* | Pyrosequencing 16S rRNA gene V1-V2 region | (Li et al., 2017a) |
| *Cow* | *Milk, colostrum, teat apex, teat canal* | *Microbiota of the bovine udder: microbiota of different compartments of udder are likely interconnected and influenced by biotic and abiotic factors such as host-associated factors (physiological and anatomical parameters, genetic traits), antimicrobial use, diet, parity and stage of lactation, management practices (housing management and milking hygiene); potential implications for udder health and mastitis susceptibility are discussed* | | | (Derakhshani et al., 2018a) |

| **Host** | **Sample** | **Aim and main result** | **Major taxa** | **Methodology** | **Reference** |
| --- | --- | --- | --- | --- | --- |
| *Human* | *Milk* | *The human milk microbiome and factors influencing its composition and activity* | | | (Gomez-Gallego et al., 2016) |
| *Human* |  | *Variation in the human milk microbiome may be related with maternal weight, mode of delivery, lactation state, gestation age, antibiotic use, and maternal health, but also milk constituents (e.g., fatty acids and complex carbohydrates)* | | | (McGuire and McGuire, 2015) |

**Table S4.** Role of milk and mammary gland microbiota for mother and infant health

| **Host** | **Sample** | **Aim and main result** | **Major taxa** | **Methodology** | **Reference** |
| --- | --- | --- | --- | --- | --- |
| **Milk microbiota and mother health** | | | | | |
| Human | Mammary tissue | Characterization of the microbiome in mammary tissue of women with or without cancer | *Bacillus, Acinetobacter, Enterobacteriaceae, Pseudomonas, Staphylococcus, Propionibacterium, Comamonadaceae, Gammaproteobacteria, Prevotella, Listeria welshimeri* | Ion Torrent 16S rRNA gene V6 region | (Urbaniak et al., 2014) |
| Human | Milk | Human milk contains a complex microbial metagenome including genomes of bacteria, archaea, viruses, fungi, and protozoa. Loss of bacterial diversity and a high increase of the sequences related to the presumptive etiological agents were observed in mastitis cases. | healthy milk: *Staphylococcus, Streptococcus, Bacteroides, Faecalibacterium, Ruminococcus, Lactobacillus, Propionibacterium.* | Shotgun pyrosequencing | (Jiménez et al., 2015) |
| Cow | Milk | Identification of potential infectious organisms in subclinical mastitis, and resistance of organisms to antibiotics and chemical compounds | Subclinical mastitis samples: *Escherichia coli* and *Staphylococcus aureus* | Shotgun pyrosequencing | (Bhatt et al., 2012) |
| Cow | Milk | Milk microbiota in relation to health status (healthy, sub-clinically and clinically diseased quarters) | healthy samples: *Propionibacterium, Aeribacillus,* unclassified *Lachnospiraceae, Faecalibacterium, Bacteroides,* unclassified *Clostridiales, Staphylococcus, Streptococcus, Anaerococcus*, unclassified *Xanthomonadaceae,* unclassified *Bacteroidales, Lactobacillus, Porphyromonas, Comamonas, Fusobacterium, Enterococcus,* unclassified *Carnobacteriaceae, Asticcacaulis* | Pyrosequencing 16S rRNA gene V1-V2 region | (Oikonomou et al., 2014) |
| Cow | Milk | Microbial profile of milk in mastitic vs healthy milk, comparison with cultural approaches | Healthy milk: *Propionibacterium,* unclassified *Ruminococcaceae, Streptococcus, Staphylococcus, Bacteroides, Fusobacterium*, | Pyrosequencing 16S rRNA gene V1-V2 region | (Oikonomou et al., 2012) |
| **Host** | **Sample** | **Aim and main result** | **Major taxa** | **Methodology** | **Reference** |
| Cow | Milk | Microbial profile of milk in mastitic vs healthy milk, comparison with cultural approaches | *Ralstonia, Pseudomonas, Sphingomonas, Stenotrophomonas, Psychrobacter, Bradyrhizobium, Corynebacterium, Pelomonas, Staphylococcus* | Pyrosequencing 16S rRNA gene V1-V2 region | (Kuehn et al., 2013) |
| Cow | Foremilk and teat canal | Reduced diversity and significant changes in teat microbiome in quarters with a history of mastitis, notably a lower *Clostridia/Bacilli* class ratio | *Staphylococcus, Corynebacterium, Ruminococcus, Aerococcus, Bifidobacterium, Flacklamia, Jeotgalicoccus, Trichococcus, Oscillospira, Lachnospiraceae (Butyrivibrio, Dorea, Roseburia), Bacteroidetes(Bacteroides, Prevotella,* | Pyrosequencing 16S rRNA gene V3-V4 region | (Falentin et al., 2016) |
| Cow | Colostrum | Impact of lactation number on colostrum microbiome; Taxonomic profile and diversity of primiparous colostrum microbiome in relation to clinical mastitis in the first month post-partum. | *Staphylococcus, Prevotella, Ruminococcaceae, Bacteroidales, Clostridiales, Pseudomonas*. | Illumina  16S rRNA gene V4 region | (Lima et al., 2017) |
| **Milk microbiota and newborn gastrointestinal microbiota** | | | | | |
| Human | Mother milk and infant mouth and gut | Shared OTUs between mother's milk and infant's mouth and gastrointestinal microbiota, including OTUs assigned to *Bifidobacterium Streptococcus* and *Staphylococcus*. Baby’s mouth could play a role in both the gastrointestinal microbiota assembly, via deglutition, and mother’s milk duct colonization, during suction. | *Streptococcus, Bifidobacterium, Staphylococcus, Lachnospiraceae, Ruminococcaceae, Bacteroidaceae* | Illumina  16S rRNA gene V3-V4 region | (Biagi et al., 2017) |
| Human | milk | During the first 30 days of life, breastfed infants received a mean of 27.7% of the bacteria from milk and 10.3% from areolar skin | milk: Proteobacteria (*Moraxellaceae, Enterobacteriaceae,* and *Pseudomonadaceae*);  areolar skin: Firmicutes (*Staphylococcaceae* and *Streptococcaceae*) | Illumina  16S rRNA gene V4 region | (Pannaraj et al., 2017) |

| **Host** | **Sample** | **Aim and main result** | **Major taxa** | **Methodology** | **Reference** |
| --- | --- | --- | --- | --- | --- |
| Human | Milk and infant feces between 1w and 3 mo | 207 genera identified in milk samples. Genera shared between infant feces and human milk samples accounted for 70–88% of the total relative abundance in infant faecal samples, and identical strains of *Bifidobacterium breve* and *Lactobacillus plantarum* were isolated from the milk and feces of one mother-infant pair, which supports the hypothesis of vertical transfer of bacteria from milk to the infant gastrointestinal tract | *Pseudomonas, Staphylococcus Streptococcus, Elizabethkingia, Variovorax, Bifidobacterium, Flavobacterium, Lactobacillus, Stenotrophomonas, Brevundimonas, Chryseobacterium and Enterobacter.* | Illumina  16S rRNA gene V3-V4 region | (Murphy et al., 2017) |
| Human | *Milk, colostrum, infant and mother feces at 1 mo and 6 mo* | mothers contribute to the infant gastrointestinal  microbiota’s resistome and mobilome development by sharing genes from their gastrointestinal and milk bacteria. | *Milk and colostrum: Streptococcus, Staphylococcus, Rothia, Lactobacillus* | Shotgun Illumina sequencing | (Pärnänen et al., 2018) |
| Human | Milk, oral cavity and feces between 2 days and 6 months | Association between milk microbiome and infant oral and fecal microbiomes and mother fecal microbiome. Data support the hypothesis that variation in the milk microbiome may influence the infant GI microbiome | *Milk: Streptococcus, Staphylococcus, Gemella, Rothia, Veillonella, Lactobacillus, Pseudomonas, Lactobacillales, Propionibacterium, Corynebacterium* | Illumina  16S rRNA gene V1-V3 region | (Williams et al., 2019) |
| *Human* |  | *Human milk beneficially influences neonatal gastrointestinal microbiota establishment by providing commensal maternal bacteria as well as a broad range of oligosaccharides that promote the growth and activity of specific bacterial populations, such as Bifidobacterium and Bacteroides spp* | | | (Jost et al., 2015) |

Aakko, J., Kumar, H., Rautava, S., Wise, A., Autran, C., Bode, L., et al. (2017). Human milk oligosaccharide categories define the microbiota composition in human colostrum. *Benef Microbes* 8, 563–567. doi:10.3920/BM2016.0185.

Addis, M. F., Tanca, A., Uzzau, S., Oikonomou, G., Bicalho, R. C., and Moroni, P. (2016). The bovine milk microbiota: insights and perspectives from -omics studies. *Molecular BioSystems*, 1–29. doi:10.1039/C6MB00217J.

Bhatt, V. D., Ahir, V. B., Koringa, P. G., Jakhesara, S. J., Rank, D. N., Nauriyal, D. S., et al. (2012). Milk microbiome signatures of subclinical mastitis-affected cattle analysed by shotgun sequencing. *Journal of applied microbiology* 112, 639–50. doi:10.1111/j.1365-2672.2012.05244.x.

Biagi, E., Quercia, S., Aceti, A., Beghetti, I., Rampelli, S., Turroni, S., et al. (2017). The Bacterial Ecosystem of Mother’s Milk and Infant’s Mouth and Gut. *Front Microbiol* 8, 1214. doi:10.3389/fmicb.2017.01214.

Boix-Amoròs, A., Collado, M. C., and Mira, A. (2016). Relationship between milk microbiota, bacterial load, macronutrients and human cells during lactation. *Frontiers in microbiology* 7, 492. doi:10.3389/fmicb.2016.00492.

Braem, G., De Vliegher, S., Verbist, B., Heyndrickx, M., Leroy, F., and De Vuyst, L. (2012). Culture-independent exploration of the teat apex microbiota of dairy cows reveals a wide bacterial species diversity. *Vet. Microbiol.* 157, 383–390. doi:10.1016/j.vetmic.2011.12.031.

Cabrera-Rubio, R., Collado, M. C., Laitinen, K., Salminen, S., Isolauri, E., and Mira, A. (2012). The human milk microbiome changes over lactation and is shaped by maternal weight and mode of delivery. *Am J Clin Nutr* 96, 544–551. doi:10.3945/ajcn.112.037382.

Catozzi, C., Sanchez Bonastre, A., Francino, O., Lecchi, C., De Carlo, E., Vecchio, D., et al. (2017). The microbiota of water buffalo milk during mastitis. *PloS one* 12, e0184710. doi:10.1371/journal.pone.0184710.

Chaves Lopez, C., Serio, A., Rossi, C., Mazzarrino, G., Marchetti, S., Castellani, F., et al. (2016). Effect of diet supplementation with Ascophyllum nodosum on cow milk composition and microbiota. *J. Dairy Sci.* 99, 6285–6297. doi:10.3168/jds.2015-10837.

Cremonesi, P., Ceccarani, C., Curone, G., Severgnini, M., Pollera, C., Bronzo, V., et al. (2018). Milk microbiome diversity and bacterial group prevalence in a comparison between healthy Holstein Friesian and Rendena cows. *Plos One* 13, e0205054. doi:10.1371/journal.pone.0205054.

Curone, G., Filipe, J., Cremonesi, P., Trevisi, E., Amadori, M., Pollera, C., et al. (2018). What we have lost: Mastitis resistance in Holstein Friesians and in a local cattle breed. *Research in Veterinary Science* 116, 88–98. doi:10.1016/j.rvsc.2017.11.020.

Derakhshani, H., Fehr, K. B., Sepehri, S., Francoz, D., De Buck, J., Barkema, H. W., et al. (2018a). Invited review: Microbiota of the bovine udder: Contributing factors and potential implications for udder health and mastitis susceptibility. *J. Dairy Sci.* doi:10.3168/jds.2018-14860.

Derakhshani, H., Plaizier, J. C., De Buck, J., Barkema, H. W., and Khafipour, E. (2018b). Composition of the teat canal and intramammary microbiota of dairy cows subjected to antimicrobial dry cow therapy and internal teat sealant. *J. Dairy Sci.* doi:10.3168/jds.2018-14858.

Dolci, P., De Filippis, F., La Storia, A., Ercolini, D., and Cocolin, L. (2014). rRNA-based monitoring of the microbiota involved in Fontina PDO cheese production in relation to different stages of cow lactation. *Int. J. Food Microbiol.* 185, 127–135. doi:10.1016/j.ijfoodmicro.2014.05.021.

Doyle, C. J., Gleeson, D., O’Toole, P. W., and Cotter, P. D. (2017). Impacts of Seasonal Housing and Teat Preparation on Raw Milk Microbiota: a High-Throughput Sequencing Study. *Appl. Environ. Microbiol.* 83. doi:10.1128/AEM.02694-16.

Drago, L., Toscano, M., De Grandi, R., Grossi, E., Padovani, E. M., and Peroni, D. G. (2017). Microbiota network and mathematic microbe mutualism in colostrum and mature milk collected in two different geographic areas: Italy versus Burundi. *ISME J* 11, 875–884. doi:10.1038/ismej.2016.183.

Falentin, H., Rault, L., Nicolas, A., Bouchard, D. S., Lassalas, J., Lamberton, P., et al. (2016). Bovine Teat Microbiome Analysis Revealed Reduced Alpha Diversity and Significant Changes in Taxonomic Profiles in Quarters with a History of Mastitis. *Frontiers in Microbiology* 7. doi:10.3389/fmicb.2016.00480.

Fernández, L., Langa, S., Martín, V., Maldonado, A., Jiménez, E., Martín, R., et al. (2013). The human milk microbiota: Origin and potential roles in health and disease. *Pharmacological Research* 69, 1–10. doi:10.1016/j.phrs.2012.09.001.

Fitzstevens, J. L., Smith, K. C., Hagadorn, J. I., Caimano, M. J., Matson, A. P., and Brownell, E. A. (2016). Systematic Review of the Human Milk Microbiota. *Nutr Clin Pract*. doi:10.1177/0884533616670150.

Frétin, M., Martin, B., Rifa, E., Isabelle, V.-M., Pomiès, D., Ferlay, A., et al. (2018). Bacterial community assembly from cow teat skin to ripened cheeses is influenced by grazing systems. *Sci Rep* 8, 200. doi:10.1038/s41598-017-18447-y.

Ganda, E. K., Bisinotto, R. S., Lima, S. F., Kronauer, K., Decter, D. H., Oikonomou, G., et al. (2016). Longitudinal metagenomic profiling of bovine milk to assess the impact of intramammary treatment using a third-generation cephalosporin. *Sci Rep* 6, 37565. doi:10.1038/srep37565.

Ganda, E. K., Gaeta, N., Sipka, A., Pomeroy, B., Oikonomou, G., Schukken, Y. H., et al. (2017). Normal milk microbiome is reestablished following experimental infection with Escherichia coli independent of intramammary antibiotic treatment with a third-generation cephalosporin in bovines. *Microbiome* 5, 74. doi:10.1186/s40168-017-0291-5.

Gill, J. J., Sabour, P. M., Gong, J., Yu, H., Leslie, K. E., and Griffiths, M. W. (2006). Characterization of bacterial populations recovered from the teat canals of lactating dairy and beef cattle by 16S rRNA gene sequence analysis. *FEMS Microbiol. Ecol.* 56, 471–481. doi:10.1111/j.1574-6941.2006.00091.x.

Gomez-Gallego, C., Garcia-Mantrana, I., Salminen, S., and Collado, M. C. (2016). The human milk microbiome and factors influencing its composition and activity. *Semin Fetal Neonatal Med* 21, 400–405. doi:10.1016/j.siny.2016.05.003.

Hunt, K. M., Foster, J. A., Forney, L. J., Schutte, U. M. E., Beck, D. L., Abdo, Z., et al. (2011). Characterization of the diversity and temporal stability of bacterial communities in human milk. *PloS one* 6, e21313. doi:10.1371/journal.pone.0021313.

Jeurink, P. V., van, B. J., Jimenez, E., Knippels, L. M., Fernandez, L., Garssen, J., et al. (2013). Human milk: a source of more life than we imagine. *Benef.Microbes.* 4, 17–30. doi:10.3920/BM2012.0040.

Jiménez, E., De Andrés, J., Manrique, M., Pareja-Tobes, P., Tobes, R., Martínez-Blanch, J. F., et al. (2015). Metagenomic analysis of milk of healthy and mastitis-suffering women. *Journal of Human Lactation* 31, 406–415. doi:10.1177/0890334415585078.

Jost, T., Lacroix, C., Braegger, C., and Chassard, C. (2013). Assessment of bacterial diversity in breast milk using culture-dependent and culture-independent approaches. *Br.J.Nutr.* 110, 1253–1262. doi:10.1017/S0007114513000597.

Jost, T., Lacroix, C., Braegger, C., and Chassard, C. (2015). Impact of human milk bacteria and oligosaccharides on neonatal gut microbiota establishment and gut health. *Nutr. Rev.* 73, 426–437. doi:10.1093/nutrit/nuu016.

Jost, T., Lacroix, C., Braegger, C. P., Rochat, F., and Chassard, C. (2014). Vertical mother-neonate transfer of maternal gut bacteria via breastfeeding. *Environmental Microbiology* 16, 2891–2904. doi:10.1111/1462-2920.12238.

Kable, M. E., Srisengfa, Y., Laird, M., Zaragoza, J., McLeod, J., Heidenreich, J., et al. (2016). The Core and Seasonal Microbiota of Raw Bovine Milk in Tanker Trucks and the Impact of Transfer to a Milk Processing Facility. *MBio* 7. doi:10.1128/mBio.00836-16.

Kuehn, J. S., Gorden, P. J., Munro, D., Rong, R., Dong, Q., Plummer, P. J., et al. (2013). Bacterial community profiling of milk samples as a means to understand culture-negative bovine clinical mastitis. *PLoS.One.* 8, e61959. doi:10.1371/journal.pone.0061959.

Kumar, H., du Toit, E., Kulkarni, A., Aakko, J., Linderborg, K. M., Zhang, Y., et al. (2016). Distinct Patterns in Human Milk Microbiota and Fatty Acid Profiles Across Specific Geographic Locations. *Front Microbiol* 7, 1619. doi:10.3389/fmicb.2016.01619.

Li, S.-W., Watanabe, K., Hsu, C.-C., Chao, S.-H., Yang, Z.-H., Lin, Y.-J., et al. (2017a). Bacterial Composition and Diversity in Breast Milk Samples from Mothers Living in Taiwan and Mainland China. *Front Microbiol* 8, 965. doi:10.3389/fmicb.2017.00965.

Li, Z., Wright, A.-D. G., Yang, Y., Si, H., and Li, G. (2017b). Unique Bacteria Community Composition and Co-occurrence in the Milk of Different Ruminants. *Scientific Reports* 7, 40950. doi:10.1038/srep40950.

Lima, S. F., Teixeira, A. G. V., Lima, F. S., Ganda, E. K., Higgins, C. H., Oikonomou, G., et al. (2017). The bovine colostrum microbiome and its association with clinical mastitis. *J. Dairy Sci.* 100, 3031–3042. doi:10.3168/jds.2016-11604.

Martín, R., Jiménez, E., Olivares, M., Marín, M. L., Fernández, L., Xaus, J., et al. (2006). Lactobacillus salivarius CECT 5713, a potential probiotic strain isolated from infant feces and breast milk of a mother-child pair. *Int. J. Food Microbiol.* 112, 35–43. doi:10.1016/j.ijfoodmicro.2006.06.011.

Martín, V., Maldonado-Barragán, A., Moles, L., Rodriguez-Baños, M., Campo, R. D., Fernández, L., et al. (2012). Sharing of bacterial strains between breast milk and infant feces. *J Hum Lact* 28, 36–44. doi:10.1177/0890334411424729.

McGuire, M. K., and McGuire, M. A. (2015). Human milk: mother nature’s prototypical probiotic food? *Adv Nutr* 6, 112–123. doi:10.3945/an.114.007435.

McInnis, E. a., Kalanetra, K. M., Mills, D. a., and Maga, E. a. (2015). Analysis of raw goat milk microbiota: Impact of stage of lactation and lysozyme on microbial diversity. *Food Microbiology* 46, 121–131. doi:10.1016/j.fm.2014.07.021.

Metzger, S. A., Hernandez, L. L., Skarlupka, J. H., Suen, G., Walker, T. M., and Ruegg, P. L. (2018). Influence of sampling technique and bedding type on the milk microbiota: Results of a pilot study. *Journal of Dairy Science* 101, 6346–6356. doi:10.3168/jds.2017-14212.

Murphy, K., Curley, D., O’Callaghan, T. F., O’Shea, C.-A., Dempsey, E. M., O’Toole, P. W., et al. (2017). The Composition of Human Milk and Infant Faecal Microbiota Over the First Three Months of Life: A Pilot Study. *Sci Rep* 7, 40597. doi:10.1038/srep40597.

Oikonomou, G., Bicalho, M. L., Meira, E., Rossi, R. E., Foditsch, C., Machado, V. S., et al. (2014). Microbiota of cow’s milk; distinguishing healthy, sub-clinically and clinically diseased quarters. *PloS one* 9, e85904. doi:10.1371/journal.pone.0085904.

Oikonomou, G., Machado, V. S., Santisteban, C., Schukken, Y. H., and Bicalho, R. C. (2012). Microbial Diversity of Bovine Mastitic Milk as Described by Pyrosequencing of Metagenomic 16s rDNA. *PLoS ONE* 7, e47671. doi:10.1371/journal.pone.0047671.

Pannaraj, P. S., Li, F., Cerini, C., Bender, J. M., Yang, S., Rollie, A., et al. (2017). Association Between Breast Milk Bacterial Communities and Establishment and Development of the Infant Gut Microbiome. *JAMA Pediatr* 171, 647–654. doi:10.1001/jamapediatrics.2017.0378.

Pärnänen, K., Karkman, A., Hultman, J., Lyra, C., Bengtsson-Palme, J., Larsson, D. G. J., et al. (2018). Maternal gut and breast milk microbiota affect infant gut antibiotic resistome and mobile genetic elements. *Nat Commun* 9, 3891. doi:10.1038/s41467-018-06393-w.

Perez, P. F., Doré, J., Leclerc, M., Levenez, F., Benyacoub, J., Serrant, P., et al. (2007). Bacterial imprinting of the neonatal immune system: lessons from maternal cells? *Pediatrics* 119, e724-32. doi:10.1542/peds.2006-1649.

Quigley, L., O’Sullivan, O., Stanton, C., Beresford, T. P., Ross, R. P., Fitzgerald, G. F., et al. (2013). The complex microbiota of raw milk. *FEMS microbiology reviews* 37, 664–98. doi:10.1111/1574-6976.12030.

Sam Ma, Z., Guan, Q., Ye, C., Zhang, C., Foster, J. A., and Forney, L. J. (2015). Network analysis suggests a potentially “evil” alliance of opportunistic pathogens inhibited by a cooperative network in human milk bacterial communities. *Sci Rep* 5, 8275. doi:10.1038/srep08275.

Soto Del Rio, M. de L. D., Dalmasso, A., Civera, T., and Bottero, M. T. (2017). Characterization of bacterial communities of donkey milk by high-throughput sequencing. *Int. J. Food Microbiol.* 251, 67–72. doi:10.1016/j.ijfoodmicro.2017.03.023.

Toscano, M., De Grandi, R., Peroni, D. G., Grossi, E., Facchin, V., Comberiati, P., et al. (2017). Impact of delivery mode on the colostrum microbiota composition. *BMC Microbiol.* 17, 205. doi:10.1186/s12866-017-1109-0.

Treven, P., Mrak, V., Bogovič Matijašić, B., Horvat, S., and Rogelj, I. (2015). Administration of probiotics Lactobacillus rhamnosus GG and Lactobacillus gasseri K7 during pregnancy and lactation changes mouse mesenteric lymph nodes and mammary gland microbiota. *J. Dairy Sci.* 98, 2114–2128. doi:10.3168/jds.2014-8519.

Urbaniak, C., Angelini, M., Gloor, G. B., and Reid, G. (2016). Human milk microbiota profiles in relation to birthing method, gestation and infant gender. *Microbiome* 4, 1. doi:10.1186/s40168-015-0145-y.

Urbaniak, C., Cummins, J., Brackstone, M., Macklaim, J. M., Gloor, G. B., Baban, C. K., et al. (2014). Microbiota of human breast tissue. *Appl. Environ. Microbiol.* 80, 3007–3014. doi:10.1128/AEM.00242-14.

Verdier-Metz, I., Gagne, G., Bornes, S., Monsallier, F., Veisseire, P., Delbès-Paus, C., et al. (2012). Cow teat skin, a potential source of diverse microbial populations for cheese production. *Applied and environmental microbiology* 78, 326–33. doi:10.1128/AEM.06229-11.

Williams, J. E., Carrothers, J. M., Lackey, K. A., Beatty, N. F., Brooker, S. L., Peterson, H. K., et al. (2019). Strong Multivariate Relations Exist Among Milk, Oral, and Fecal Microbiomes in Mother-Infant Dyads During the First Six Months Postpartum. *J. Nutr.* doi:10.1093/jn/nxy299.

Williams, J. E., Carrothers, J. M., Lackey, K. A., Beatty, N. F., York, M. A., Brooker, S. L., et al. (2017). Human milk microbial community structure is relatively stable and related to variations in macronutrient and micronutrient intakes in healthy lactating women. *The Journal of nutrition* 147, 1739–1748. doi:10.3945/jn.117.248864.

Young, W., Hine, B. C., Wallace, O. A. M., Callaghan, M., and Bibiloni, R. (2015). Transfer of intestinal bacterial components to mammary secretions in the cow. *PeerJ* 3, e888. doi:10.7717/peerj.888.

Zhang, R., Huo, W., Zhu, W., and Mao, S. (2015). Characterization of bacterial community of raw milk from dairy cows during subacute ruminal acidosis challenge by high-throughput sequencing. *J. Sci. Food Agric.* 95, 1072–1079. doi:10.1002/jsfa.6800.
